# Multifaceted regulation of sirtuin 2 (Sirt2) deacetylase activity

**DOI:** 10.1016/j.jbc.2024.107722

**Published:** 2024-08-28

**Authors:** Maheeshi Yapa Abeywardana, Samuel D. Whedon, Kwangwoon Lee, Eunju Nam, Rafael Dovarganes, Sarah DuBois-Coyne, Ishraq A. Haque, Zhipeng A. Wang, Philip A. Cole

**Affiliations:** 1 Division of Genetics, Department of Medicine, Brigham and Women’s Hospital, Boston, Massachusetts, USA; 2 Department of Biological Chemistry and Molecular Pharmacology, Harvard Medical School, Boston, Massachusetts, USA; 3 Desai Sethi Urology Institute & Sylvester Comprehensive Cancer Center, University of Miami Miller School of Medicine, Miami, Florida, USA

**Keywords:** protein semisynthesis,, enzymes, nucleosomes, phosphorylation, histones

## Abstract

Sirtuin 2 (Sirt2) is a member of the sirtuin family of NAD-dependent lysine deacylases and plays important roles in regulation of the cell cycle and gene expression. As a nucleocytoplasmic deacetylase, Sirt2 has been shown to target both histone and nonhistone acetylated protein substrates. The central catalytic domain of Sirt2 is flanked by flexible N and C termini, which vary in length and composition with alternative splicing. These termini are further subject to posttranslational modifications including phosphorylation. Here, we investigate the function of the N and C termini on deacetylation of nuclear substrates by Sirt2. Remarkably, we find that the C terminus autoinhibits deacetylation, while the N terminus enhances deacetylation of proteins and peptides, but not nucleosomes—a chromatin model substrate. Using protein semisynthesis, we characterize the effect of cell cycle-linked N-terminal phosphorylation at two major phosphorylation sites (Ser23/Ser25) and find that these further enhance protein/peptide deacetylation, with no effect on nucleosome deacetylation. Additionally, we find that VRK1, an established binding partner of both Sirt2 and nucleosomes, can stimulate deacetylation of nucleosomes by Sirt2, likely through an electrostatic mechanism. Taken together, these findings reveal multiple mechanisms regulating the activity of Sirt2, which allow for a broad range of activities across its multiple biological roles.

The sirtuin family of NAD-dependent lysine deacetylases (1) have been linked to multiple facets of cellular physiology, including metabolism (2), cell division (3), DNA repair (4), and gene expression (5). Sirtuin 2 (Sirt2) is a nucleocytoplasmic sirtuin, which is believed to act on nuclear histones (6), transcription factors, and ATP citrate lyase (7), among others. Sirt2 is recruited to the nucleus during mitosis (2) where it is believed to catalyze the deacetylation of chromatin on various histone lysine (Lys) sites (8). Sirt2 is hypothesized to serve as a tumor suppressor (9) or a driver of malignancy depending on context. Inhibitors of Sirt2 exhibit anticancer activity in preclinical models (10, 11).

Sirt2 is a 389 amino acid protein comprised of a core deacetylase domain flanked by flexible N (aa1-65) and C termini (aa 340–389) (12), which have been shown to be subject to posttranslational modifications (13). Phosphorylation sites in the N and C termini have been mapped in multiple proteomic studies (14) (https://www.phosphosite.org/proteinAction.action?id=19822&showAllSites=true). Several mRNA splice variants of Sirt2 (15) are reported, one of which omits amino acid residues 1 to 37, and thus three of the most prominent phosphorylation sites (16). Nonetheless, there is limited functional characterization (17, 18) of the Sirt2 N and C terminal tails beyond indirect evidence from cellular studies (6). Enzymological studies have generally used truncated crystallography constructs (aa56–356 or aa38–356) with acetylated peptide substrates (19, 20). To clarify the nuclear functions of Sirt2, we focused on physiologically relevant acetylated nucleosome substrates and nuclear interacting partners like the kinase VRK1 (21). Nucleosomes, the basic building blocks of chromatin, consist of a histone octamer that contains two copies each of histones H2A, H2B, H3, and H4 wrapped by ∼147 bp of DNA (22). Recent studies have shown distinct site-selectivities among various acyltransferases (23) and deacylases (24) when comparing nucleosome *versus* peptide or free histone protein substrates.

We analyze how the N and C termini of Sirt2, as well as interaction with VRK1, influence deacetylation of nucleosome and non-nucleosome substrates by Sirt2. In addition, we use protein semisynthesis to generate site-specifically phosphorylated Sirt2 to explore its effect on catalysis. Taken together, these experiments reveal multiple mechanisms of regulating Sirt2 enzymatic activity.

## Results

### Deacetylase activity of full-length and truncated Sirt2 forms

**Figure 1:**
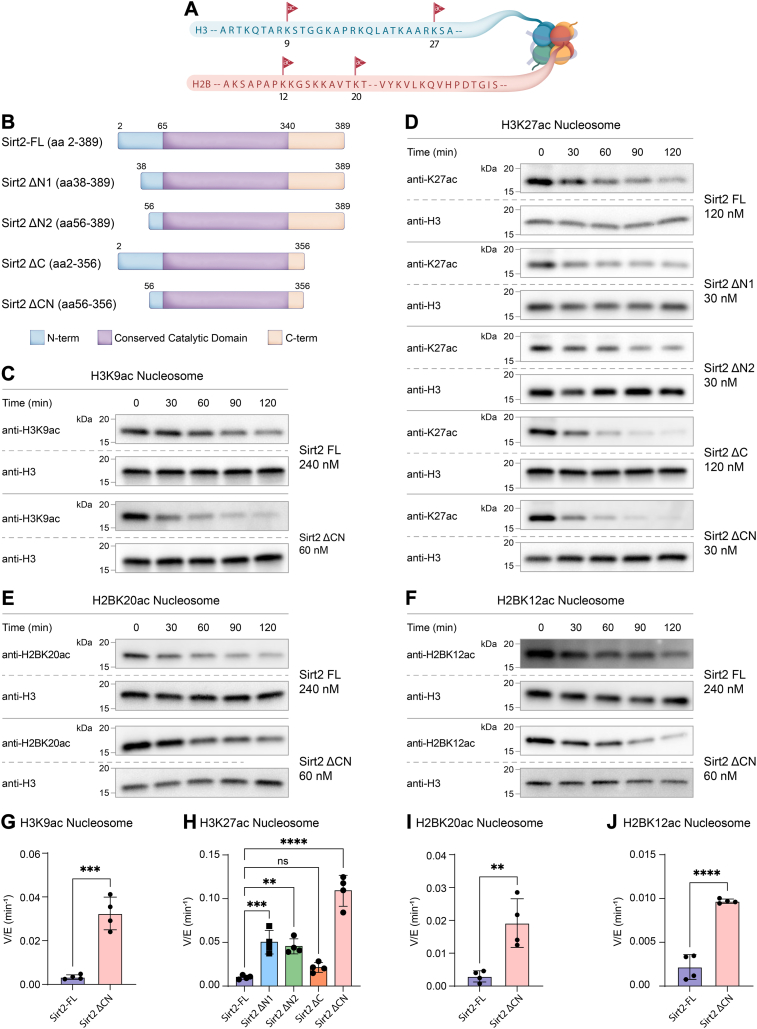
**Sirt2 deacetylation on H3 nucleosome sites.***A*, illustration of N-terminal tail acetylation sites of histone H3 and H2B used in this manuscript. *B*, illustration of different Sirt2 protein constructs used in this manuscript. *C*, Western blot analysis of H3K9ac nucleosome deacetylation by Sirt2-FL and Sirt2 ΔCN protein constructs. *D*, Western blot analysis of H3K27ac nucleosome deacetylation by different Sirt2 protein constructs. *E*, Western blot analysis of H2BK20ac nucleosome deacetylation by Sirt2-FL and Sirt2 ΔCN protein constructs. *F*, Western blot analysis of H2BK12ac nucleosome deacetylation by Sirt2-FL and Sirt2 ΔCN protein constructs. *G*, comparison of V/[E] for H3K9ac nucleosome deacetylation by Sirt2-FL and Sirt2 ΔCN protein constructs. Error bars represent SEM of at least three independent experiments. Statistical significance was calculated using unpaired *t* test ∗∗∗*p* < 0.0002. *H*, bar graphs representing the comparison of V/[E] for H3K27ac nucleosome deacetylation by different Sirt2 protein constructs. Error bars represent SEM of at least three independent experiments. Statistical analysis was conducted using one-way ANOVA followed by Tukey’s *post hoc* test ∗∗*p* < 0.002, ∗∗∗*p* < 0.0002, ∗∗∗∗*p* < 0.0001. *I*, comparison of V/[E] for H2BK20ac nucleosome deacetylation by Sirt2-FL and Sirt2 ΔCN protein constructs. Error bars represent SEM of at least three independent experiments. Statistical significance was calculated using unpaired *t* test ∗∗*p* < 0.002. *J*, comparison of V/[E] for H2BK12ac nucleosome deacetylation by Sirt2-FL and Sirt2 ΔCN protein constructs. Error bars represent SEM of at least three independent experiments. Statistical significance was calculated using unpaired *t* test ∗∗∗∗*p* < 0.0001. Sirt2, sirtuin 2; Sirt2-FL, full-length Sirt2.

**Figure 2:**
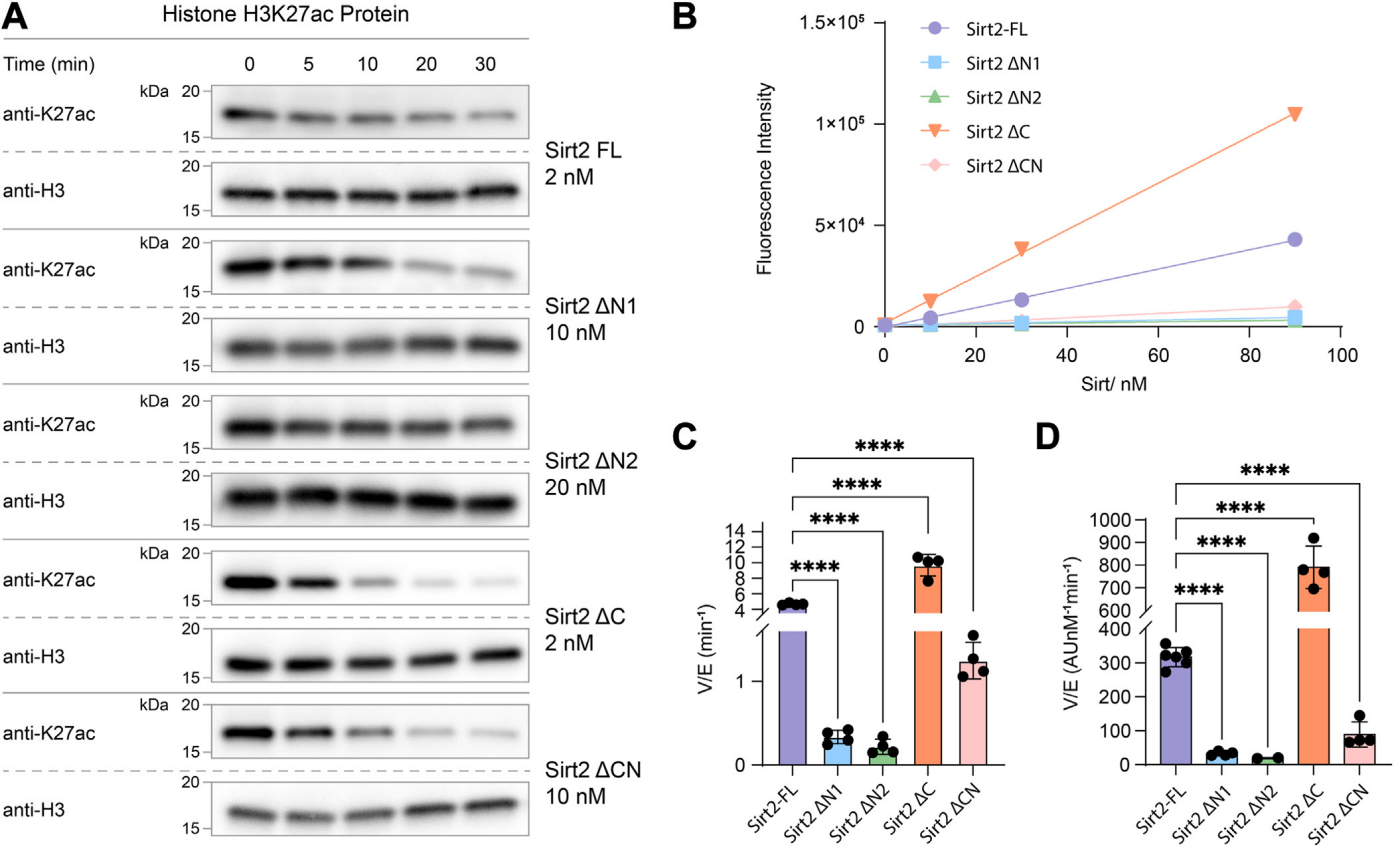
**Sirt2 deacetylation on histone H3K27ac protein and peptide.***A*, Western blot analysis of histone H3K27ac protein deacetylation by different Sirt2 constructs. *B*, Fluor de Lys assay of acetylated peptide deacetylation by Sirt2 protein constructs. *C*, bar graphs representing V/[E] comparisons of histone H3K27ac protein deacetylation by different Sirt2 protein constructs. Error bars represent SEM of at least three independent experiments. Statistical significance was calculated using unpaired *t* test followed by Tukey’s *post hoc* test ∗∗∗∗*p* < 0.0001. *D*, bar graphs representing V/[E] comparisons of acetylated peptide deacetylation by different Sirt2 protein constructs. Error bars represent SEM of at least two independent experiments. Statistical analysis was conducted using one-way ANOVA followed by Tukey’s *post hoc* test ∗∗∗∗*p* < 0.0001. Sirt2, sirtuin 2; Sirt2-FL, full-length Sirt2.

Full-length and truncated recombinant human tag-free Sirt2 proteins were expressed and purified from *E. coli* and appeared monomeric (25) by size-exclusion chromatography (26) (Figs. S1 and S2). Deacetylation assays were conducted using nucleosomes containing 147 bp DNA and site-specific acetylation at H3K9, H3K27, H2BK12, or H2BK20. The commonly used Sirt2 core construct, aa56 to 356 (Sirt2 ΔCN), exhibited ∼3-fold more rapid deacetylation of H3K27ac nucleosomes (V/[E] = 0.09 min^−^^1^) than of H3K9ac nucleosomes (V/[E] = 0.03 min^−^^1^) (Figs. 1, S5*E* and S6*B*, and Table S2). In contrast, full-length Sirt2 (aa1-389, Sirt2-FL) (V/[E] = 0.009 min^−1^) displayed a ∼10-fold rate reduction in deacetylation of H3K27ac nucleosomes and a proportional drop in the deacetylation rates for H3K9ac (V/[E] = 0.003 min^−1^), H2BK12ac, and H2BK20ac (Figs. 1, Fig. S5*A*, S6*A*, and S7, and Table S2). We hypothesized that Sirt2-FL was autoinhibited as a catalyst by one or both of its N and C termini and thus carried out deacetylase assays with a short peptide substrate as well as free histone H3K27ac protein to investigate this. Unexpectedly, full-length Sirt2 was ∼3-fold more active at deacetylating both peptide and H3K27ac protein (V/[E] = 4.61 min^−1^) compared with the aa56 to 356 truncated Sirt2 form (V/[E] = 1.28 min^−1^) (Fig. 2, Fig. S8, *A* and *E*, S11*A*, and Table S4). These results indicate that deletion of the N and C termini of Sirt2 does not lead to a general catalytic enhancement but one that is nucleosome specific (Fig. 1).

To gain further insight into the role of the N and C termini in Sirt2 enzymatic activity, we prepared the purified single segment deletion Sirt2 proteins aa1-356 (Sirt2 ΔC), aa56 to 389 (Sirt2 ΔN2) as well as the isoform 2 splice variant aa38 to 389 (Sirt2 ΔN1) (Fig. S1 and S2). Removing the N-terminal segments aa1-55 or aa1-37 from Sirt2 accelerated H3K27ac nucleosome deacetylation 4-fold relative to Sirt2-FL (Fig. 1, *D* and *H*, Fig.S5, and Table S1). Likewise, deletion of the C terminus (aa1-356) accelerated deacetylation of H3K27ac nucleosomes ∼2-fold (V/[E] = 0.02 min^−^^1^) relative to Sirt2-FL (Fig. 1, *D* and *H*, Fig.S5, and Table S1). Thus, each of the unstructured termini of Sirt2-FL contributes to impeding nucleosome deacetylation, with a greater effect from the N terminus compared to the C terminus (Fig. 1).

In contrast to the results with nucleosome substrates, N-terminally truncated Sirt2 (aa56–389) displayed slower deacetylation of peptide and histone protein substrates, while C-terminally truncated Sirt2 (aa1-356) showed faster peptide and histone protein substrate deacetylation (Fig. 2, Fig. S8 and S11*A*, Tables S4 and S5). These results indicate that removal of the Sirt2 C terminus generally enhances deacetylase activity in a substrate-independent fashion. The N terminus, however, shows more complex behavior as it accelerates peptide substrate deacetylation but antagonizes nucleosome deacetylation. In spite of these varied effects on catalytic rate, the measured Km for NAD was fairly similar across all constructs (Fig. S16), suggesting that differential NAD-binding affinity is unlikely to account for differences in catalytic activity.

### VRK1 impact on Sirt2 deacetylase activity

**Figure 3:**
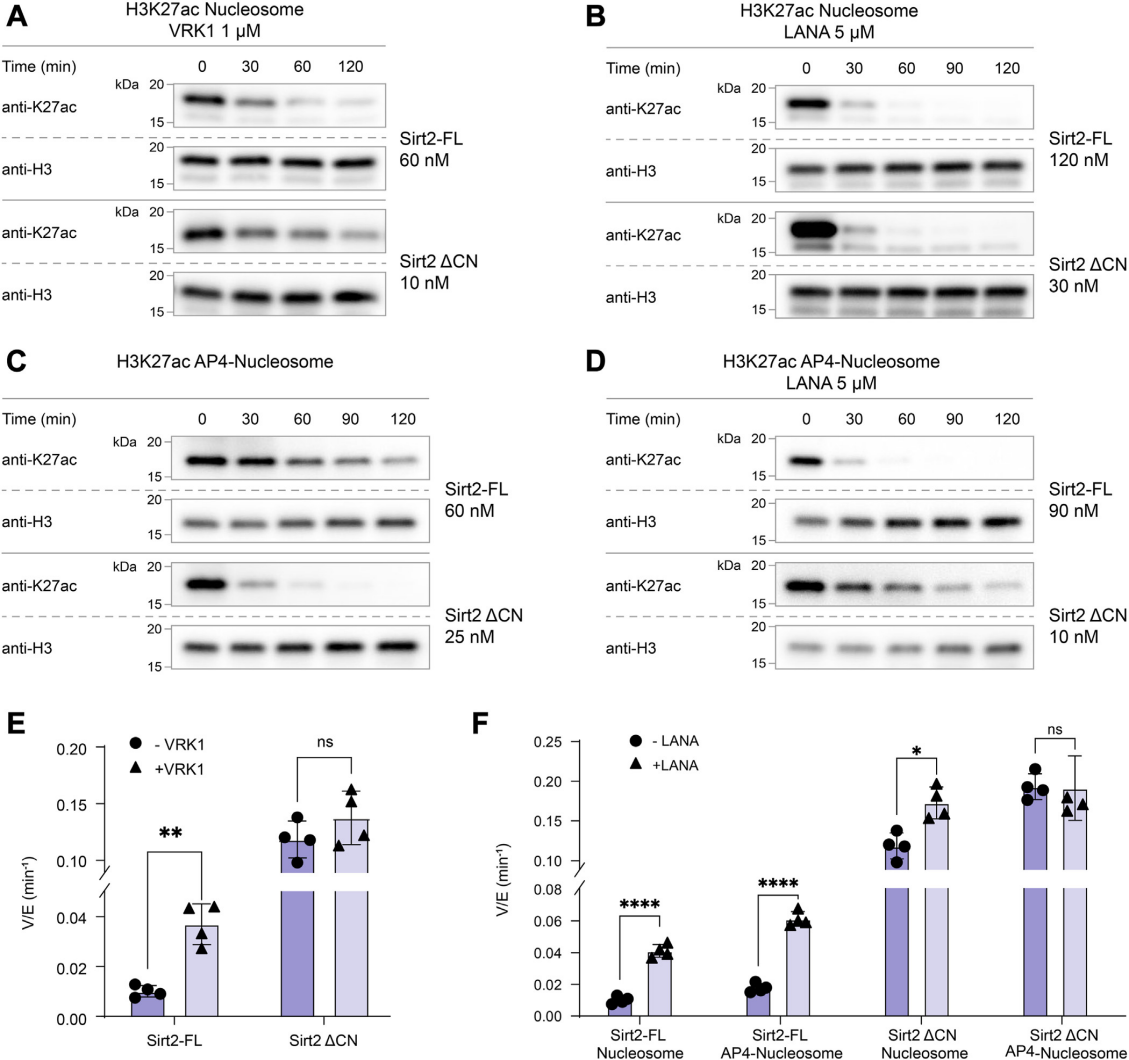
**H3K27ac nucleosome deacetylation by Sirt2 proteins in presence of VRK1 protein and LANA peptide.***A*, Western blot analysis of H3K27ac nucleosome deacetylation by Sirt2-FL and Sirt2 ΔCN protein constructs in the presence of VRK1 protein. *B*, Western blot analysis of H3K27ac nucleosome deacetylation by Sirt2-FL and Sirt2 ΔCN protein constructs in the presence of LANA peptide. *C*, Western blot analysis of H3K27ac AP4-Nucleosome (H2A E61A/E64A/D90A/E92A) deacetylation by Sirt2-FL and Sirt2 ΔCN protein constructs. *D*, Western blot analysis of H3K27ac AP4-Nucleosome deacetylation by Sirt2-FL and Sirt2 ΔCN protein constructs in the presence of LANA peptide. *E*, bar graphs representing the comparison of V/[E] for H3K27ac nucleosome deacetylation by different Sirt2 protein constructs. Error bars represent SEM of at least three independent experiments. Statistical analysis was conducted using multiple *t* test, ∗∗*p* < 0.001. *F*, bar graphs representing the comparison of V/[E] for H3K27ac nucleosome and AP4-Nucleosomes deacetylation by different Sirt2 protein constructs. Error bars represent SEM of at least 3 independent experiments. Statistical analysis was conducted using multiple *t* test, ∗*p* < 0.03, ∗∗∗∗*p* < 0.0001. Sirt2, sirtuin 2; Sirt2-FL, full-length Sirt2.

Prior studies have suggested a direct interaction between VRK1 and Sirt2, which inhibits VRK1’s kinase activity (27). As VRK1 is a nucleosome-binding protein with unstructured N and C termini (28) and a probable Sirt2-binding partner (21), we tested Sirt2 deacetylase activity in the presence of purified full-length VRK1 protein. VRK1 increased full-length Sirt2 deacetylase activity ∼3-fold toward H3K27ac nucleosome substrate, with a slight dependence on VRK1 concentration (1 μM VRK1, V/[E] = 0.03 min^-1^) about 3.5-fold faster, whereas 300 nM VRK1 showed a 2.5-fold stimulation (Fig. 3, *A* and *E*, Fig. S12, *A*, *B*, and *E*, and Table S3). Conversely, truncated Sirt2 (aa56–356) exhibited a modest increase of ∼1.4-fold with 1 μM VRK1 (Fig. 3, *A* and *C*, Fig. S12, *C*–*E*, and Table S3). Using a peptide substrate Sirt2-FL and Sirt2 aa56 to 356 deacetylation was unaffected by VRK1 (Fig. S11*F*), suggesting that stimulation of Sirt2 by VRK1 may involve nucleosome-specific contacts.

### Novel semisynthesis of site-specifically diphosphorylated Sirt2

**Figure 4:**
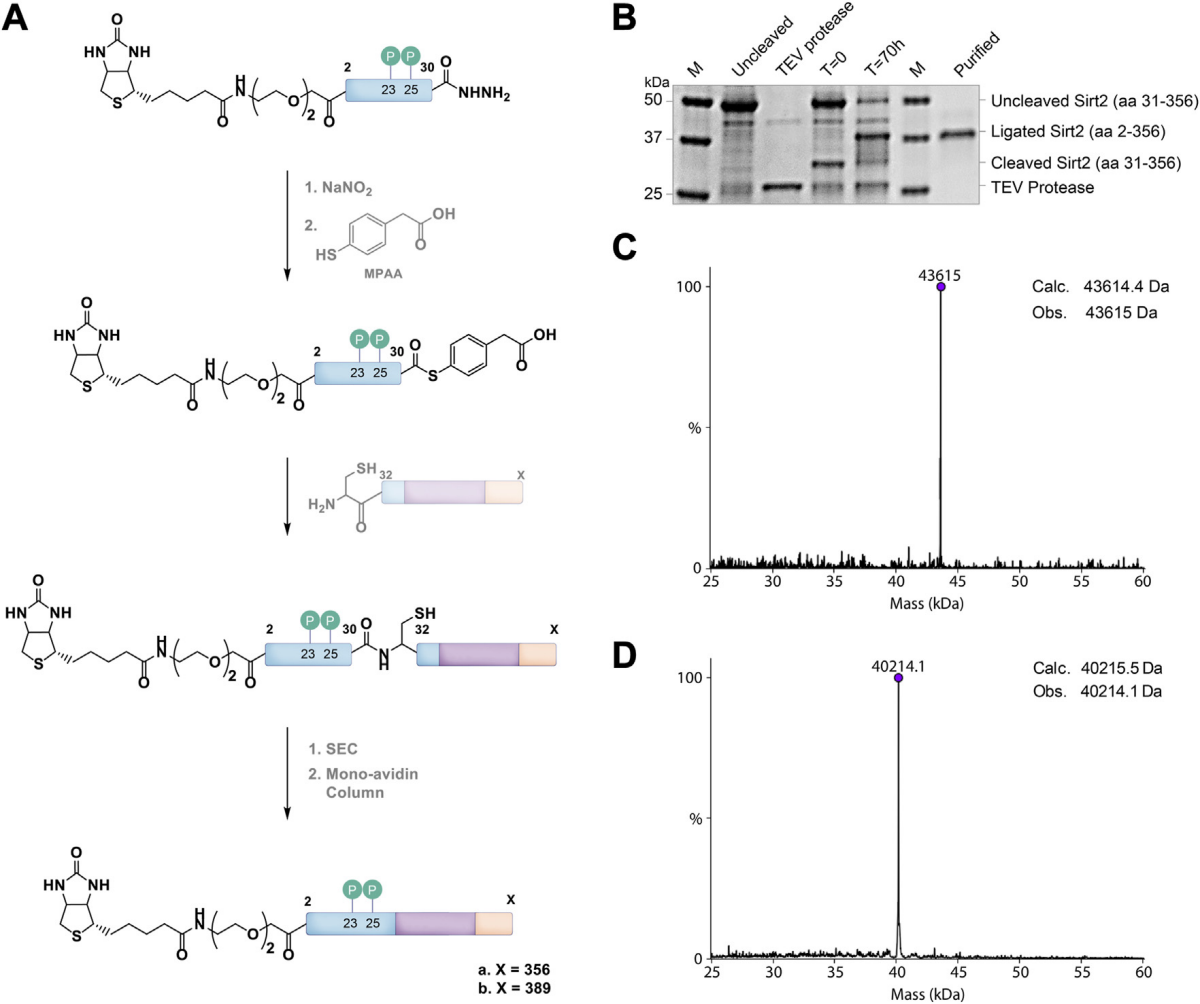
**Generation of diphosphorylated Sirt2 protein constructs.***A*, scheme for native chemical ligation to generate full-length and ΔC Sirt2 protein constructs with site-specific phosphorylation at Ser23 and Ser25 as well as a biotin at the N terminus for the purification. *B*, SDS-PAGE analysis of the ligation. *C* and *D*, deconvoluted ESI-MS of diphosphorylated Sirt2-FL and diphosphorylated Sirt2 ΔC, respectively, after ligation. Sirt2, sirtuin 2; Sirt2-FL, full-length Sirt2.

Phosphorylation on Ser23 and Ser25 in the N terminus of Sirt2 have been observed in numerous mass-spectrometry–based phosphoproteomic studies (14, 29, 30). Moreover, phosphorylation of Ser25 has been suggested to have the functional consequence of reducing the association between Sirt2 and chromatin (13). Given the varied effects of N and C termini on catalytic activity, we applied protein semisynthesis to produce Ser23/Ser25 dually phosphorylated Sirt2. In this approach, a Sirt2 A31C (aa31–389) fragment was produced recombinantly with an N-terminal Cys exposed after proteolytic removal of a His6 tag. A Sirt2 aa1-30 peptide containing a C-terminal thioester, phosphorylations at Ser23 and Ser25, and an N-terminal biotin was produced using solid phase peptide synthesis *via* the oxidation of a C-terminal peptide hydrazide (Fig. 4*A*) (31). Chemoselective ligation of the peptide and protein was achieved (Fig. 4*B*), and the full-length semisynthetic diphosphorylated Sirt2 (diphospho-Sirt2) was purified to near homogeneity and confirmed by mass spectrometry (Fig. 4, *C* and *D*, Fig. S1*F* and S3).

### Analysis of semisynthetic site-specifically diphosphorylated Sirt2

**Figure 5:**
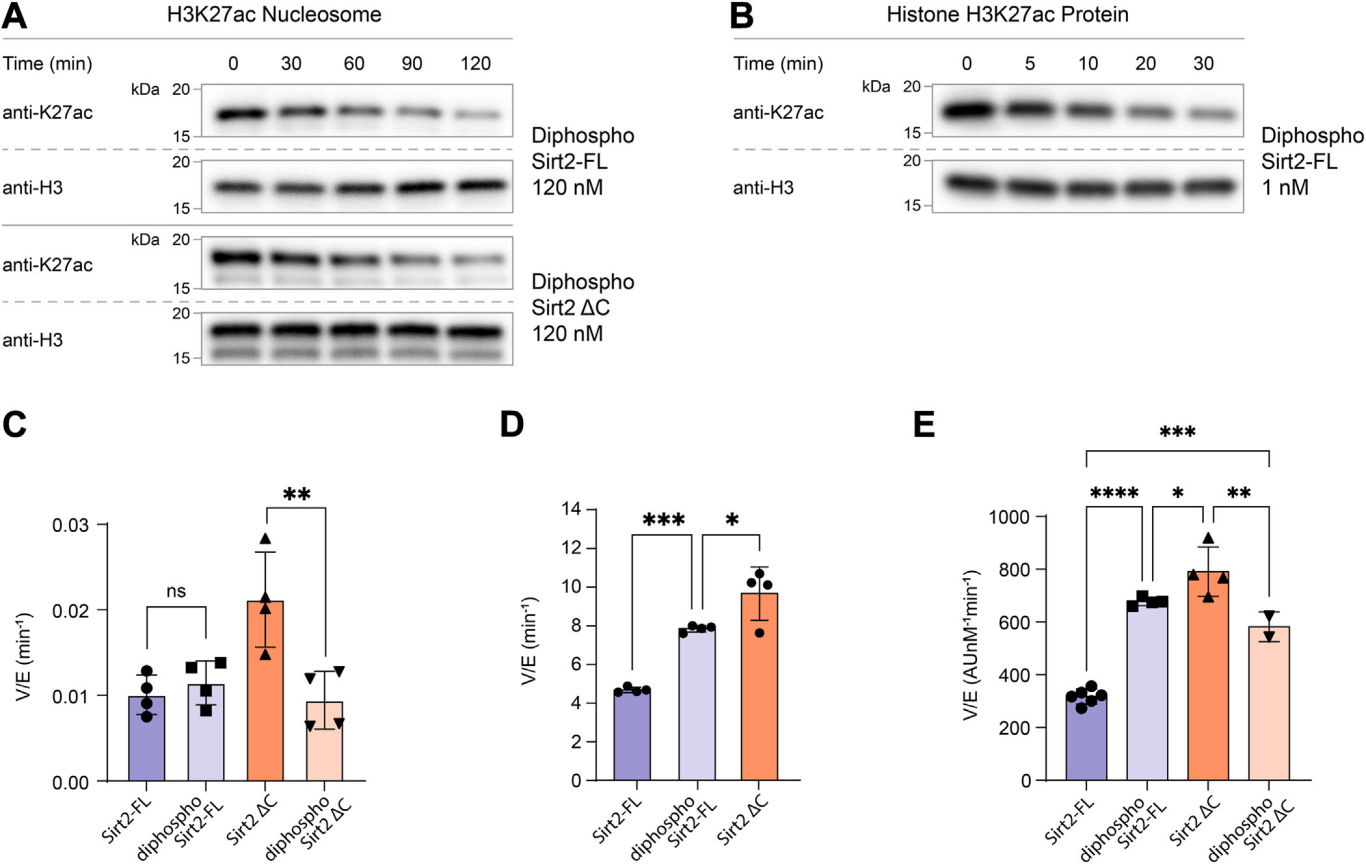
**H3K27ac nucleosome deacetylation by diphosphorylated Sirt2 proteins.***A*, Western blot analysis of H3K27ac nucleosome deacetylation by diphosphorylated Sirt2-FL and diphosphorylated Sirt2 ΔC protein constructs. *B*, Western blot analysis of histone H3K27ac protein deacetylation by diphosphorylated Sirt2-FL. *C*, bar graphs representing the comparison of V/[E] for H3K27ac nucleosome deacetylation by different Sirt2 protein constructs. Error bars represent SEM of at least three independent experiments. Statistical analysis was conducted using one-way ANOVA followed by Tukey’s *post hoc* test, ∗*p* < 0.03, ∗∗*p* < 0.002. *D*, bar graphs representing the comparison of V/[E] for histone H3K27ac protein deacetylation by different Sirt2 protein constructs. Error bars represent SEM of at least three independent experiments. Statistical analysis was conducted using one way ANOVA followed by Tukey’s *post hoc* test, ∗*p* < 0.03, ∗∗∗*p* < 0.0002. *E*, bar graphs representing the comparison of V/[E] for acetylated peptide deacetylation by different Sirt2 protein constructs. Error bars represent SEM of at least two independent experiments. Statistical analysis was conducted using one-way ANOVA followed by Tukey’s *post hoc* test, ∗*p* < 0.03, ∗∗*p* < 0.002, ∗∗∗*p* < 0.0002, ∗∗∗∗*p* < 0.0001. Sirt2, sirtuin 2; Sirt2-FL, full-length Sirt2.

Diphospho-Sirt2 was next assayed for H3K27ac nucleosome deacetylase activity and found to have a similar catalytic rate to that of full-length, recombinant, and unmodified Sirt2 (Fig. 5, *A* and *C*, Fig.S9*A*, and Table S1). Assays with peptide or protein substrate, however, revealed that diphospho-Sirt2 showed ∼3-fold greater deacetylase activity compared with the unmodified recombinant protein (Fig. 5, *B*, *D* and *E*, Fig. S10, S11*B*, Tables S4, and S5). To confirm that this was due to phosphorylation and not A31C mutation, we treated diphospho-Sirt2 with lambda phosphatase to remove the N-terminal phosphorylations. Re-assay of the dephosphorylated protein revealed that its catalytic activity was now similar to that of the unmodified, full-length, and recombinant WT Sirt2 (Fig.S1*G* and S11*C*). These results establish that phosphorylation of Ser23/Ser25 is responsible for the gain of activity with the full-length semisynthetic diphospho-Sirt2 protein.

It was notable that diphospho-Sirt2 displayed a similar peptide deacetylase activity to that of the unmodified C-terminally truncated Sirt2. This similarity led us to consider that dual phosphorylation of Ser23/Ser25 might display electrostatic interactions with the positively charged C terminus in an intramolecular fashion, thereby relieving autoinhibition by the C terminus. To test this, we generated C-terminally truncated (aa1-356) semisynthetic diphospho-Sirt2 protein and assayed it with acetylated peptide substrate (Figs. S1*H* and S4). Remarkably, truncation of the C terminus resulted in similar activity to recombinant, unmodified Sirt2 as well as full-length diphospho-Sirt2 protein (Fig. 5*E*, Fig.S11*B*, and Table S5). These results are consistent with the idea that dual phosphorylation of the N terminus relieves autoinhibition by the C terminus through an electrostatic interaction. However, C-terminally truncated (aa1-356) semisynthetic diphospho-Sirt2 was slower than the equivalent unphosphorylated enzyme at H3K27ac nucleosome deacetylation (Fig. 5, *A* and *C*, Fig. S9*B*, and Table S1).

### Deacetylase activity of full-length and truncated Sirt2 forms with increased ionic strength

**Figure 6:**
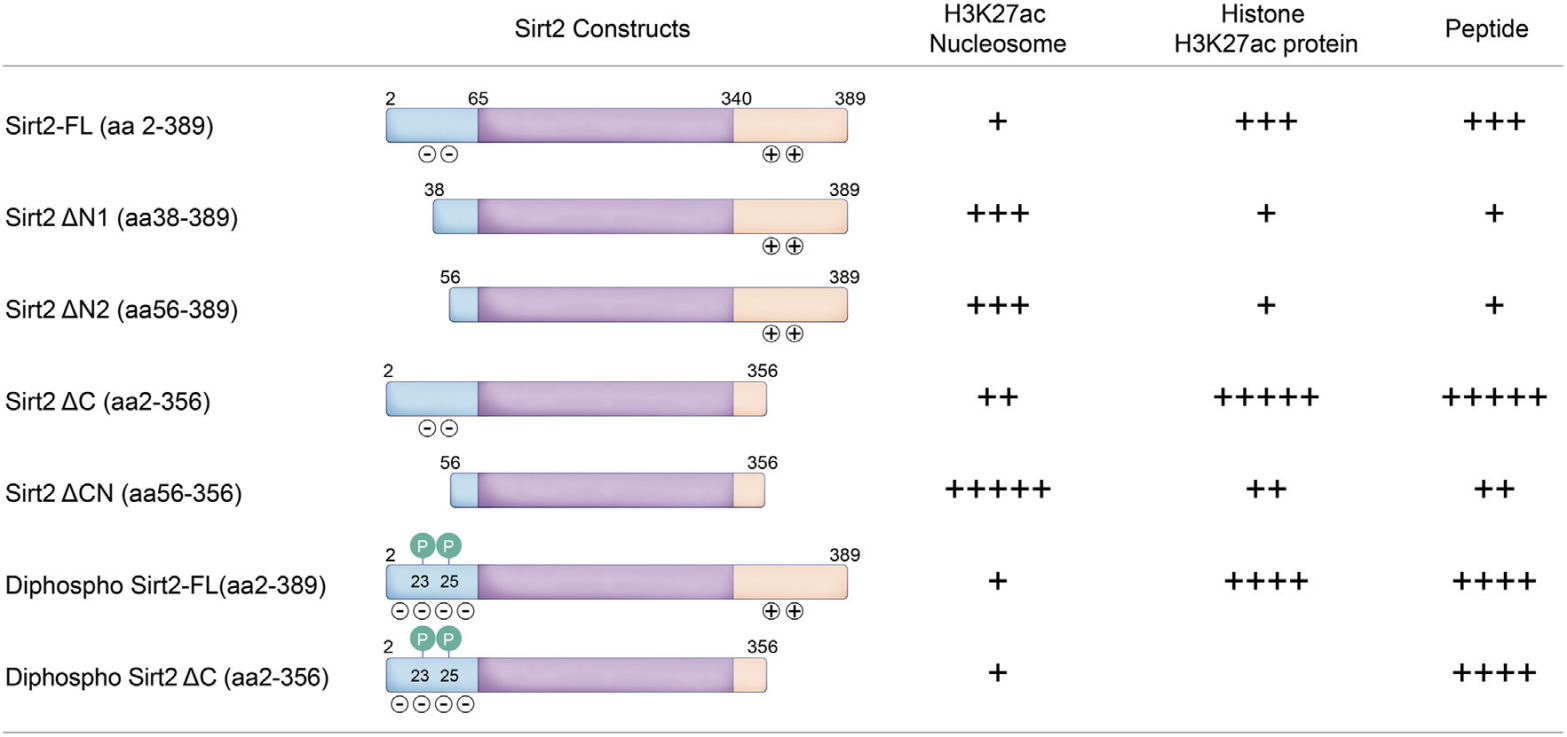
**Comparison of the Sirt2’s catalytic activity toward different substrates by different Sirt2 constructs.** Sirt2, sirtuin 2; Sirt2-FL, full-length Sirt2.

**Figure 7:**
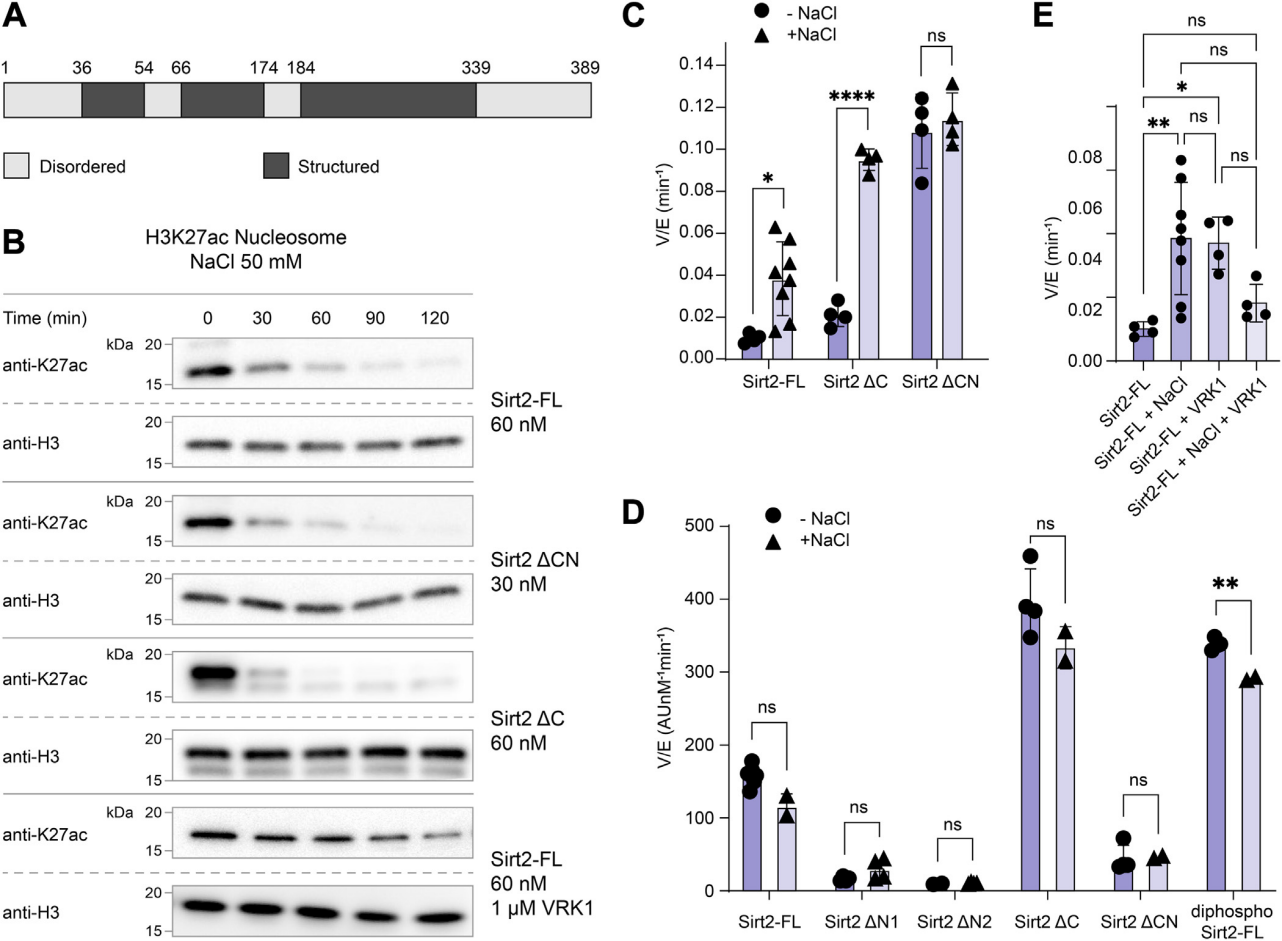
**H3K27ac nucleosome deacetylation by Sirt2 proteins in presence of 50 mM NaCl.***A*, schematic of the disordered regions of Sirt2 by bioinformatic prediction (PONDR). The N-terminal domain (aa1-36) and C-terminal domain (aa339–389) predicted to be disordered. *B*, Western blot analysis of H3K27ac nucleosome deacetylation by Sirt2-FL, Sirt2 ΔCN, Sirt2 ΔC, and Sirt2-FL with VRK1 protein constructs in presence of 50 mM NaCl. *C*, bar graphs representing the comparison of V/[E] for H3K27ac nucleosome deacetylation by different Sirt2 protein constructs. Error bars represent SEM of at least three independent experiments. Statistical analysis was conducted using multiple *t* test, ∗*p* < 0.03 and ∗∗∗∗*p* < 0.0001. *D*, bar graph representing the comparison of V/[E] for peptide deacetylation by different Sirt2 protein constructs. Error bars represent SEM of at least two independent experiments. Statistical analysis was conducted using multiple *t* test, ∗∗*p* < 0.002. E, comparison of V/[E] for H3K27ac nucleosome deacetylation by Sirt2-FL protein. Error bars represent SEM of at least three independent experiments. Statistical significance was calculated using one way ANOVA followed by Tukey’s *post hoc* test ∗*p* < 0.03, ∗∗*p* < 0.002. Sirt2, sirtuin 2; Sirt2-FL, full-length Sirt2.

Sequence analysis showed that both the N and C termini are largely unstructured (Fig. 6*A*), leading us to hypothesize that the electrostatic interactions involved in Sirt2 binding to the nucleosome influences the interactions between its own N and C termini. To further understand the distinct influence of the N and C termini, as well as diphosphorylation on the deacetylase activity of Sirt2 toward nucleosome and peptide substrates, we tested activity in the presence of more NaCl. The addition of 50 mM NaCl significantly accelerated deacetylation of H3K27ac nucleosomes by Sirt2-FL and the C-terminally truncated Sirt2, but not the N- and C-terminally truncated Sirt2 (Fig. 7, *B* and *C*, Fig. S14, and Table S3). However, salt did not affect peptide-based deacetylation rate of any of the Sirt2 constructs (Fig. 7*D* and Fig. S11*E*). We also explored the effect of VRK1 on Sirt2-FL-catalyzed nucleosome in the presence of added NaCl. Notably, 50 mM NaCl addition abolished stimulation by VRK1 observed with low salt (Figs. 7*E* and S15). These results reveal a key electrostatic autoinhibition mediated by the N terminus of Sirt2 that can be mitigated either by the presence of VRK1 or elevated NaCl concentration.

### Role of the nucleosome H2A/H2B acidic patch in Sirt2-catalyzed deacetylation

Many enzymes that act on nucleosomes, including Sirt6, exploit interactions with the histone H2A/histone H2B acidic patch for efficient catalysis. In addition, VRK1 is known to interact with the nucleosome acidic patch. We thus attempted to examine the effects of the H2A/H2B acidic patch in nucleosome deacetylation of Sirt2 by employing a synthetic LANA peptide, derived from the KSHV LANA protein, which is known to have high affinity for the H2A/H2B acidic patch in nucleosomes (29). Interestingly, LANA peptide addition enhanced H3K27ac nucleosome deacetylation by full-length Sirt2 with only a minor rate effect on Sirt2 aa56 to 356 (Fig. 3, *A*, *B* and *F*, Fig. S13, and Table S3). The impact was similar to that of VRK1.

To explore the LANA peptide effect further, we tested mutant H3K27ac nucleosomes in which two or four mutations in the histone H2A acidic residues were replaced with Ala (AP2-nucleosome: H2A D90A/E92A and AP4-nucleosome: H2A E61A/E64A/D90A/E92A). These H2A acidic patch nucleosomes showed small stimulation of both full-length Sirt– and truncated Sirt2 (aa56–356)–mediated nucleosome deacetylation (Fig. 3, *C* and *F*, Fig. S17, S18, and Table S6). Much to our surprise, LANA peptide-related effects on Sirt2 deacetylation were minimally impacted by these acidic patch mutations (Fig 3, *D* and *F*, Fig. S17, and Table S6). These results suggest that LANA stimulation of Sirt2-nucleosome acetylation is likely not occurring through binding the nucleosome acidic patch.

## Discussion

In this study, we have uncovered several features regulating Sirt2 catalyzed deacetylation of nucleosome and non-nucleosome substrates. Previous studies have tacitly treated the Rossman fold domain as the full-length protein, focusing primarily on the catalytic domain and active site (32). Thus, attempting to recapitulate the deacetylation activity of Sirt2 *in vitro*, these studies did not probe how the termini might affect enzymatic function. These mechanisms may be particularly critical in the context of complex protein and nucleosomal substrates and protein-binding partners. Recent studies have shown that nucleosomes may, in some cases, be more biologically relevant substrates of sirtuins (20).

Notably, the action of Sirt2 on nucleosome substrates is autoinhibited by its N and C termini. Sirt2 has been reported to deacetylate several acetyl-Lys sites in nucleosomes including H3K9ac (33), H3K18ac (34), and H4K16ac (35) based on cellular studies. In this study, we assessed four sites across histone H3 and H2B and showed that the inhibitory influences and the N- and C-terminal tails were fairly similar (within 2-fold) at each of these positions. These results suggest that the autoinhibition may not lead to altered Sirt2 site specificity. The molecular basis for the N- and C-terminal autoinhibition of Sirt2 deacetylation appears to involve two distinct mechanisms. The C terminus generally inhibits Sirt2 catalysis, regardless of substrate (peptide or nucleosome). In contrast, the N terminus selectively inhibits nucleosome deacetylation, while promoting peptide and free histone H3 deacetylation. How the N terminus antagonizes nucleosome deacetylation by Sirt2 is unresolved but may stem from electrostatic repulsion between its high Asp/Glu content and negatively charged surfaces on nucleosomal DNA. Furthermore, the fact that 50 mM NaCl addition appears to relieve the autoinhibition mediated by the Sirt2 N terminus supports this idea. The total ionic strength of the enzyme reaction solution containing 50 mM NaCl (which includes 50 mM Hepes) is in the same range of physiological buffer which raises the possibility that the N-terminal autoinhibition of nucleosome deacetylation, and the influence of VRK1, may not be significant in normal cellular conditions. In contrast to Sirt2, nucleosome deacetylation by the sirtuin Sirt6 is highly inhibited by NaCl addition (24), revealing the diversity of the catalytic behaviors across the enzyme family. We note, however, that Sirt2’s C-terminal region is autoinhibitory for nucleosome deacetylation at lower and higher salt concentration. Moreover, with peptide substrate, changes in the ionic strength did not appear to influence regulation by both the Sirt2 N- and C-terminal segments, pointing to the physiological importance of these interactions on non-nucleosome substrates.

In Sirt2, there appears to be crosstalk between the N and C termini. Phosphorylation of Ser23 and Ser25, which activates Sirt2 on peptide substrates, matches the catalytic effects of C-terminal truncation. Furthermore, removing the C terminus in the context of N-terminal phosphorylation does not further augment peptide deacetylase activity. Our proposed model for this crosstalk is direct intramolecular interaction between the N and C termini mediated by electrostatic interactions, which relieves the presumed autoinhibition caused by the C terminus. The overall amino acid sequence of the N terminus is quite acidic, intensified by Ser phosphorylation, whereas the C terminus is largely basic. Deletion of the N terminus appears to facilitate autoinhibition by the C terminus. Furthermore, phosphorylation of the N terminus attenuates autoinhibition by the C terminus. This result is consistent with previous modeling research (18) on the autoinhibitory function of the C terminus and with non-nucleosome–based *in vitro* assays. Taken together, these data support a key role for electrostatics in regulating Sirt2.

The influence of N-terminal phosphorylation on Sirt2 deacetylase activity toward nucleosomes is less straightforward to interpret. Phosphorylation of the N terminus of Sirt2-FL showed no detectable effect on deacetylation of H3K27ac nucleosomes. Mechanistic insight came from assaying C-terminally truncated (aa1-356), semisynthetic diphospho-Sirt2 with H3K27ac nucleosomes. In this case, C-terminal deletion of diphospho-Sirt2 did not stimulate nucleosome deacetylation as it did with unphosphorylated Sirt2. Our model to explain these results is that N-terminal Ser23/Ser25 phosphorylation of Sirt2 shows offsetting effects. We propose that Ser23/Ser25 phosphorylation attenuates C-terminal autoinhibition which in isolation would enhance Sirt2’s deacetylation kinetics as it does with peptide substrate. Superimposed on this, however, is that Ser23/Ser25 may hinder Sirt2 nucleosome binding or orientation for catalysis. Hence, the net effect is little change to the nucleosome deacetylation rate (Fig. 7).

In addition, here we employ a variant of expressed protein ligation (36) that involves a peptide hydrazide precursor to a C-terminal thioester (37). Typically, this ligation method requires oxidation at – 20 °C and under denaturing conditions (6 M guanidine HCl), which are not well-tolerated with native proteins. However, in this study, we employed oxidation at 4 °C in a mild aqueous buffer, achieving nearly quantitative yield, due to the absence of oxidizable amino acid residues (Met, Tyr, and Trp). This underscores the general utility of the hydrazide ligation in native peptide and protein chemistry.

In contrast to Sirt6(25), nucleosome deacetylation by Sirt2 does not seem to have a major dependence on the nucleosome acidic patch. Our findings with LANA peptide’s positive impact on nucleosome deacetylation were initially interpreted as a large role for the acidic patch in antagonizing Sirt2-catalyzed nucleosome deacetylation. However, the use of acidic patch mutant nucleosomes contradicted this idea and suggests that LANA peptide may not always be a specific tool for examining the acidic patch in nucleosomes.

Recent reports indicate that Sirt2 catalytic domain may undergo dimerization (25) although distinct interfaces and dramatically different binding affinities have been observed (26). In this study, we have not observed evidence of dimerization by size-exclusion chromatography with any of the Sirt2 forms prepared here and under similar buffer and assay conditions. Nevertheless, it is plausible that in the presence of particular substrates, dimerization of Sirt2 may contribute to its catalytic regulation.

In summary, we have investigated the Sirt2 N- and C-terminal tails, VRK1, and N-terminal phosphorylation and their effects on Sirt2-catalyzed deacetylation nucleosome and non-nucleosome substrates. This complex set of regulatory features may drive distinct biological outputs that allow for a mosaic of Sirt2 functions in cell physiology (38).

## Experimental procedures

### Reagents

All reagents, chemicals, and solvents used in this study were of commercial grade. Fmoc-amino acids were obtained from Ambeed Inc., while coupling reagents were sourced from Sigma-Aldrich. Molecular cloning enzymes were purchased from New England Biolabs, and the original Sirt2 plasmid was generously provided by Professor Hening Lin. Antibodies were acquired as specified in the detailed methods section.

### Expression and purification of FL-Sirt2 and other Sirt2 constructs

All human Sirt2 constructs were transformed into *E. coli* BL21(DE3). Single colonies were picked and grown overnight in 10 ml LB media starter culture supplemented with 50 mg/L kanamycin and 35 mg/L chloramphenicol. The starter culture was then diluted into 1 L LB media supplemented with 50 mg/L kanamycin and 35 mg/L chloramphenicol in a shaker flask. Cells were grown with constant shaking (200 rpm) at 37 °C. After several hours when the *A*_600_ reached 0.6, cells were induced by adding 0.5 mM IPTG. The temperature was then reduced to 16 °C, and the cells were further grown for 16 h at 16 °C with continuous shaking. Following incubation, the cells were pelleted by centrifugation (4000*g*, 4°C, 15 min), and the supernatant was removed. The cell pellets were resuspended in 30 ml cold lysis buffer (20 mM Tris, 500 mM NaCl, 20 mM imidazole, and 0.5 mM TCEP, pH 7.8) and lysed by passing through a French press three times. The cell lysate was then centrifuged (12,000*g*, 4 °C, 30 min), and the supernatant was incubated for 1 h at 4 °C with preequilibrated Ni-NTA resin (2 ml resin bed volume/1 L culture). The flow-through was removed, the resin was sequentially washed with 10 column volumes of wash buffer (20 mM Tris, 500 mM NaCl, 50 mM imidazole, and 0.5 mM TCEP, pH 7.8), followed by 25 column volumes of high-salt wash buffer (20 mM Tris, 2 M NaCl, 20 mM imidazole, and 0.5 mM TCEP, pH 7.8), and then with 25 column volumes of lysis buffer. Proteins were eluted with 10 column volumes of elution buffer (20 mM Tris, 500 mM NaCl, 250 mM imidazole, and 0.5 mM TCEP, pH 7.8) and concentrated using an Amicon spin concentrator (10 kDa MWCO, 4000 rpm, 4°C). His-SUMO-Sirt2-ΔCN was mixed with UlpI protease at a mass ratio of 1:100 (UlpI: Sirt2), while other Sirt2 constructs were mixed with TEV protease at a mass ratio of 1:100 (TEV protease: Sirt2) before overnight dialysis at 4 °C in dialysis buffer containing 20 mM Tris, 150 mM NaCl, and 0.5 mM TCEP, pH 7.5 (eluted His-SUMO-TEV-Sirt2 A31C-ΔN and His-SUMO-TEV-Sirt2 A31C-ΔCN proteins were dialyzed, concentrated and directly used for the subsequent ligation reactions without further purification). Dialyzed samples were incubated with pre-equilibrated Ni-NTA resin for 1 h at 4 °C, and the flow-through was concentrated using an Amicon spin concentrator (30 kDa MWCO, 4000 rpm, 4 °C). The concentrated samples were purified by size-exclusion chromatography using a Superdex200 column (Superdex200 buffer: 50 mM Tris, 150 mM NaCl, and 0.5 mM TCEP, pH 7.5). Eluted fractions were checked by SDS-PAGE, and the purified fractions were combined and concentrated using an Amicon spin concentrator (10 kDa, 4000 rpm, 4 °C). Purity and protein concentration were determined by SDS-PAGE and densitometry. Selected Sirt2 protein forms were characterized by intact ESI-MS (Q Exactive, Thermo Scientific) and deconvoluted using UniDec software. The concentrated protein forms were aliquoted, flash-frozen in liquid nitrogen, and stored at −80 °C for further usage. Eluted His-SUMO-TEV-Sirt2 A31C-ΔCN proteins were dialyzed, concentrated, and directly used for the subsequent ligation reactions.

### Diphosphorylated N-terminal peptide synthesis

The diphosphorylated N-terminal peptide hydrazide of Sirt2 (2–30) was synthesized using standard Fmoc-based solid-phase peptide synthesis on 2-chlorotrityl chloride resin (Chem-Impex, cat# 12996) as reported previously. Fmoc-Ser(PO(OBzl)OH)-OH (2 eq, 0.4 mmol) was coupled with HATU (2 eq, 0.4 mmol) and DIPEA (3 eq, 0.6 mmol) in dimethylformamide (4 ml) by agitation for 2 h on an end-over-end rotator, followed by a dimethylformamide wash (4 ml) and a second identical overnight coupling. The coupling of Fmoc-3-amino-3,6-dioxaoctanoic acid and biotin followed the same protocol.

After been cleaved with Reagent B [2.5% triisopropylsilane, 5% water, 5% phenol, and 87.5% trifluoroacetic acid (TFA)] for 120 min on an end-over-end rotator, the crude peptide was precipitated by adding ice-cold diethyl ether (10 volumes) to the filtrate. The crude peptide was then purified using reverse-phase HPLC with a C18 semi-preparative column (Varian Dynamax Microsorb 100, 250 × 21.4 mm) with a linear gradient from 5% acetonitrile (CH3CN)/0.05% TFA to 95% CH3CN/0.05% TFA over 40 min. Purified fractions were assessed by ESI-MS (Q Exactive, Thermo Scientific), and these were combined, lyophilized, and stored at −80 °C.

### Semisynthesis of diphosphorylated Sirt2

The N-terminal diphosphorylated peptide hydrazide (aa 2–30, pS23, pS25, 3 mg) was dissolved in 0.2 M phosphate solution (pH 3.0–3.1, 270 μl) in an Eppendorf tube and incubated in a water bath at 4 °C for 15 min. The N-terminal deleted Sirt2 protein (Sirt2 A31C-ΔN or Sirt2 A31C-ΔCN, 1.3 mg) with uncleaved TEV recognition sequence was diluted in 0.2 M phosphate buffer, pH 6.8, to a final volume of 600 μl. To this solution, 4-mercaptophenylacetic acid (7.5 mg) and TEV protease were added, and the pH was adjusted to 6.8 with 6 M NaOH. The diphosphorylated peptide hydrazide was oxidized to the corresponding azide by adding 0.5 M NaNO2 (30 μl) at 4 °C and gently agitated for 5 min. After incubation for 5 min, the C-terminal Sirt2 protein solution was added to the oxidation solution dropwise, and the pH of the ligation mixture was adjusted to 6.8 to 7.0 with minimum amounts of 6 M NaOH. The reaction proceeded at 4 °C for 72 h, and the reaction progress was monitored by SDS-PAGE.

The ligated product was purified using Superdex200 size-exclusion column (SEC) chromatography with a flow rate of 0.5 ml/min in a buffer containing 50 mM Tris, 150 mM NaCl, 0.5 mM TCEP, pH 7.5, and 0.4 ml fractions were collected. Fractions containing the ligated protein were combined and further purified using mono-avidin resin. The combined SEC fractions were incubated with 1 ml of preblocked mono-avidin resin (Thermo, cat# 20267) for 30 min on ice. The flow-through was collected and incubated with the resin two more times with 30-min incubations on ice. The resin was washed with SEC buffer containing 500 mM NaCl (10 column volumes) for 1 h, followed by SEC buffer containing 1 M NaCl (10 column volumes) for 1 h, and then with SEC buffer (10 column volumes). Protein elution was performed by incubating resin with 10 mM biotin in SEC buffer (1 ml each, 6 times) for 10 to 15 min on ice. The elution fractions containing the ligated product were combined and concentrated. The purified semisynthetic protein was characterized by ESI-MS (Q Exactive, Thermo Scientific) and deconvoluted using UniDec.

### Deacetylation assay on acetylated nucleosome and histone protein substrates by Sirt2

H3K9ac and H3K27ac nucleosomes with 147 bp DNA and histone H3K27ac protein were prepared based on a previously reported protocol (24). AP2-nucleosome and AP4-nucleosome were derived from previous literature (28). The histone protein (final concentration 1 μM) or nucleosome substrates (final concentration 100 nM) were diluted in sirtuin reaction buffer (50 mM Hepes, 1 mM DTT, 0.2 mg/ml BSA, and 1 mM NAD, pH 7.5). The reactions were initiated by adding different Sirt2 constructs [Sirt2-FL (aa 2–356), Sirt2-ΔCN (aa 56–356), Sirt2-ΔN1 (aa 38–389), Sirt2-ΔN2 (aa 56–389), Sirt2-ΔC (aa 2–356), diphospho-Sirt2-FL (aa 2–389), and diphospho-Sirt2-ΔC(aa 2–356)] to different final concentrations and incubated at 37 °C. Multiple reaction samples from the same reaction tubes were taken and quenched at different time points (for nucleosome substrates, time points include 0, 30, 60, 90, and 120 min, and for protein substrates, time points include 0, 5, 10, 20, and 30 min). In a typical assay, the reaction was quenched by mixing the sample with quenching buffer (2× Laemmli sample buffer containing 20 mM EDTA) at a 1:1 ratio to a final concentration of 10 mM EDTA and 1× Laemmli sample buffer. Samples were boiled at 95 °C for 3 to 5 min and resolved on 4 to 20% SDS-PAGE gels (TGXTM, Bio-Rad, 4561096) at 180 V for 20 to 25 min. Proteins were then transferred to nitrocellulose membranes (Transfer Stack, Invitrogen, IB301031) using the iBlot (Invitrogen) transfer system with P3 (20 V) for 5 min, blocked in BSA in TBST (20 mM Tris, 150 mM NaCl, and 0.1% Tween, pH 7.5) for 1 h at room temperature, and incubated with primary antibodies [anti-H3K9ac (Abcam, cat# 32129, 1:1000 dilution), anti-H3K27ac (Cell Signaling Technology, cat# 8173S, 1:1000 dilution) for site-specific modifications, and anti-H3 (Abcam, cat# ab1791, 1:1000 dilution) for total H3] overnight at 4 °C. Membranes were washed with TBST and then incubated with HRP-linked anti-rabbit IgG secondary antibody (Cell Signaling #7074S) for 1 h at room temperature. The specificity of each antibody used was validated previously (20). Subsequently, the membranes were treated with ECL substrate (Bio-Rad, cat# 170–5061) and visualized using a G:BOX mini gel imager (Syngene). The bands on the membrane were quantified using ImageJ software. Relative band intensities were obtained by dividing the band intensities by the intensity at T = 0, and the data were fit to a single-phase exponential decay curve (GraphPad Prism 10, with constrains Y0 = 1 and plateau = 0), with each data point representing at least two replicates. The kinetic parameter (V/[E]) was calculated using GraphPad Prism 10. Furthermore, the remaining nucleosome samples after the assay were resolved on a native TBE gel (4–20%, Novex, Thermo Fisher Scientific EC62252BOX) at 120V for 110 min alongside the nucleosomes before the assay to confirm the stability of the nucleosomes during the assay.

### Fluor de Lys assay on peptide substrates by Sirt2

The Fluor de Lys assay was conducted following the manufacturer’s protocol (BPS Lifesciences, cat# 50087). Sirt2 constructs were diluted in sirtuin reaction buffer (50 mM Hepes, 1 mM DTT, 0.2 mg/ml BSA, and 1 mM NAD, pH 7.5) to prepare a 900 μM stock solution. Sirt peptide substrate was also diluted in the same buffer to obtain a stock solution of 500 μM. A final concentration of 50 μM substrate was mixed with a concentration gradient of Sirt2 (0 nM, 10 nM, 30 nM, and 90 nM) in separate wells of a black 96-well plate (MicroWellTM, Thermo, #267342). A well containing only the reaction buffer served as the negative control. The plate, covered with aluminum foil, was incubated at 37 °C for 30 min, followed by the addition of Sirt2 developer (2X) to achieve a final concentration of 1X, and further incubated for 15 min at room temperature. The fluorescence intensities of the samples were measured using a microtiter-plate reading fluorimeter (BioTek Cytation 5) with excitation at 365 nm and emission at 450 nm wavelengths. Data were analyzed by plotting linear fits after subtracting the negative control using GraphPad Prism, with at least two replicates for each data point.

### Dephosphorylation of diphosphorylated Sirt2 by lambda phosphatase

The diphosphorylated semisynthetic Sirt2 protein was incubated with lambda phosphatase at a mass ratio of 1:100 (lambda phosphatase: diphosphorylated Sirt2) for 2 h at 30 °C. The protein was then characterized by ESI-MS (Q Exactive, Thermo Scientific) and deconvoluted using UniDec. The product was further used for deacetylation assays.

## Data availability

All data are contained within the article.

## Supporting information

This article contains supporting information.

## Conflict of interest

The authors declare no conflicts of interest with the contents of this article.

## References

[1] Jing H., Lin H. (2015). Sirtuins in epigenetic regulation. Chem. Rev..

[2] Wang M., Lin H. (2021). Understanding the function of mammalian sirtuins and protein lysine acylation. Annu. Rev. Biochem..

[3] Spiegelman N.A., Zhang X., Jing H., Cao J., Kotliar I.B., Aramsangtienchai P. (2019). SIRT2 and lysine fatty acylation regulate the activity of RalB and cell migration. ACS Chem. Biol..

[4] Nguyen P., Shukla S., Liu R., Abbineni G., Smart D.K. (2019). Sirt2 regulates radiation-induced injury. Radiat. Res..

[5] Ali I., Conrad R.J., Verdin E., Ott M. (2018). Lysine acetylation goes global: from epigenetics to metabolism and therapeutics. Chem. Rev..

[6] Eldridge M.J.G., Pereira J.M., Impens F., Hamon M.A. (2020). Active nuclear import of the deacetylase Sirtuin-2 is controlled by its C-terminus and importins. Sci. Rep..

[7] Schiedel M., Herp D., Hammelmann S., Swyter S., Lehotzky A., Robaa D. (2018). Chemically induced degradation of sirtuin 2 (Sirt2) by a proteolysis targeting chimera (PROTAC) based on sirtuin rearranging ligands (SirReals). J. Med. Chem..

[8] Zhang H., Dammer E.B., Duong D.M., Danelia D., Seyfried N.T., Yu D.S. (2022). Quantitative proteomic analysis of the lysine acetylome reveals diverse SIRT2 substrates. Sci. Rep..

[9] Minten E.V., Kapoor-Vazirani P., Li C., Zhang H., Balakrishnan K., Yu D.S. (2021). SIRT2 promotes BRCA1-BARD1 heterodimerization through deacetylation. Cell Rep..

[10] Spiegelman N.A., Price I.R., Jing H., Wang M., Yang M., Cao J. (2018). Direct comparison of SIRT2 inhibitors: potency, specificity, activity-dependent inhibition, and on-target anticancer activities. ChemMedChem..

[11] Singh S., Kumar P.U., Thakur S., Kiran S., Sen B., Sharma S. (2015). Expression/localization patterns of sirtuins (SIRT1, SIRT2, and SIRT7) during progression of cervical cancer and effects of sirtuin inhibitors on growth of cervical cancer cells. Tumor Biol..

[12] Head P.E., Zhang H., Bastien A.J., Koyen A.E., Withers A.E., Daddacha W.B. (2017). Sirtuin 2 mutations in human cancers impair its function in genome maintenance. J. Biol. Chem..

[13] Pereira J.M., Chevalier C., Chaze T., Gianetto Q., Impens F., Matondo M. (2018). Infection reveals a modification of SIRT2 critical for chromatin association. Cell Rep..

[14] Mertins P., Mani D.R., Ruggles K.V., Gillette M.A., Clauser K.R., Wang P. (2016). Proteogenomics connects somatic mutations to signalling in breast cancer. Nature.

[15] Yang Y., Ding J., Gao Z.G., Wang Z.J. (2017). A variant in SIRT2 gene 3’-UTR is associated with susceptibility to colorectal cancer. Oncotarget.

[16] Ota T., Suzuki Y., Nishikawa T., Otsuki T., Sugiyama T., Irie R. (2004). Complete sequencing and characterization of 21,243 full-length human cDNAs. Nat. Genet..

[17] North B.J., Verdin E. (2007). Mitotic regulation of SIRT2 by cyclin-dependent kinase 1-dependent phosphorylation. J. Biol. Chem..

[18] Li J., Flick F., Verheugd P., Carloni P., Lüscher B., Rossetti G. (2015). Insight into the mechanism of intramolecular inhibition of the catalytic activity of Sirtuin 2 (SIRT2). PLoS One.

[19] Teng Y. Bin, Jing H., Aramsangtienchai P., He B., Khan S., Hu J. (2014). Efficient demyristoylase activity of SIRT2 revealed by kinetic and structural studies. Sci. Rep..

[20] Wang Z.A., Whedon S.D., Wu M., Wang S., Brown E.A., Anmangandla A. (2022). Histone H2B deacylation selectivity: exploring chromatin’s dark matter with an engineered sortase. J. Am. Chem. Soc..

[21] Monte-Serrano E., Morejón-García P., Campillo-Marcos I., Campos-Díaz A., Navarro-Carrasco E., Lazo P.A. (2023). The pattern of histone H3 epigenetic posttranslational modifications is regulated by the VRK1 chromatin kinase. Epigenetics Chromatin.

[22] Wang Z.A., Millard C.J., Lin C.-L., Gurnett J.E., Wu M., Lee K. (2020). Diverse nucleosome site-selectivity among histone deacetylase complexes. eLife.

[23] Weinert B.T., Narita T., Satpathy S., Srinivasan B., Hansen B.K., Schölz C. (2018). Time-resolved analysis reveals rapid Dynamics and broad scope of the CBP/p300 acetylome. Cell.

[24] Wang Z.A., Markert J.W., Whedon S.D., Yapa Abeywardana M., Lee K., Jiang H. (2023). Structural basis of sirtuin 6-catalyzed nucleosome deacetylation. J. Am. Chem. Soc..

[25] Suzuki N., Konuma T., Ikegami T., Akashi S. (2024). Biophysical insights into the dimer formation of human Sirtuin 2. Protein Sci..

[26] Yang J., Nicely N.I., Weiser B.P. (2023). Effects of dimerization on the deacylase activities of human SIRT2. Biochemistry.

[27] Monte-Serrano E., Lazo P.A. (2023). VRK1 kinase activity modulating histone H4K16 acetylation inhibited by SIRT2 and VRK-IN-1. Int. J. Mol. Sci..

[28] Budziszewski G.R., Zhao Y., Spangler C.J., Kedziora K.M., Williams M.R., Azzam D.N. (2022). Multivalent DNA and nucleosome acidic patch interactions specify VRK1 mitotic localization and activity. Nucleic Acids Res..

[29] Mertins P., Qiao J.W., Patel J., Udeshi N.D., Clauser K.R., Mani D.R. (2013). Integrated proteomic analysis of post-translational modifications by serial enrichment. Nat. Methods.

[30] Bian Y., Song C., Cheng K., Dong M., Wang F., Huang J. (2014). An enzyme assisted RP-RPLC approach for in-depth analysis of human liver phosphoproteome. J. Proteomics.

[31] Zheng J.-S., Tang S., Qi Y.-K., Wang Z.-P., Liu L. (2013). Chemical synthesis of proteins using peptide hydrazides as thioester surrogates. Nat. Protoc..

[32] Feldman J.L., Dittenhafer-Reed K.E., Kudo N., Thelen J.N., Ito A., Yoshida M. (2015). Kinetic and structural basis for Acyl-group selectivity and NAD+ dependence in sirtuin-catalyzed deacylation. Biochemistry.

[33] Vaquero A., Scher M.B., Dong H.L., Sutton A., Cheng H.L., Alt F.W. (2006). SirT2 is a histone deacetylase with preference for histone H4 Lys 16 during mitosis. Genes Dev..

[34] Eldridge M.J.G., Hamon M.A. (2021). Histone H3 deacetylation promotes host cell viability for efficient infection by Listeria monocytogenes. PLoS Pathog..

[35] Chen X., Lu W., Wu D. (2021). Sirtuin 2 (SIRT2): confusing roles in the pathophysiology of neurological disorders. Front. Neurosci..

[36] Wang Z.A., Cole P.A. (2020). Methods and applications of expressed protein ligation. Methods Mol. Biol..

[37] Muir T.W., Sondhi D., Cole P.A. (1998). Expressed protein ligation: a general method for protein engineering. Proc. Natl. Acad. Sci. USA.

[38] Head P.S.E., Kapoor-Vazirani P., Nagaraju G.P., Zhang H., Rath S.K., Luong N.C. (2023). DNA-PK is activated by SIRT2 deacetylation to promote DNA double-strand break repair by non-homologous end joining. Nucleic Acids Res..

