# Supplementary material for: Multifaceted regulation of sirtuin 2 (Sirt2) deacetylase activity
Source: J Biol Chem. 2024 Aug 28;300(9):107722. doi: 10.1016/j.jbc.2024.107722 (PMC11458557; doi:10.1016/j.jbc.2024.107722)

**Multifaceted regulation of Sirtuin 2 (Sirt2) Deacetylase Activity**

Maheeshi Yapa Abeywardana^1^, Samuel D. Whedon^1^, Kwangwoon Lee^1^, Eunju Nam^1^, Rafael Dovarganes^1^, Sarah Dubois-Coyne^1^, Ishraq A. Haque^1^, Zhipeng A. Wang,* ^1,2^ and Philip A. Cole*^1^

1. Division of Genetics, Department of Medicine, Brigham and Women’s Hospital, Boston, Massachusetts 02115, United States; Department of Biological Chemistry and Molecular Pharmacology, Harvard Medical School, Boston, Massachusetts 02115, United States;

2. Desai Sethi Urology Institute, Sylvester Comprehensive Cancer Center, University of Miami Miller School of Medicine, Miami, FL 33136, United States

**Primers**

1. Sirt2_∆N1_F

5’- ATGGATTTTCTTCGCAATC

Sirt2_∆N1_R

5’- CTGAAAATACAGGTTTTCG

2. Sirt2_∆N2_F

5’- GAGCGTTTGCTTGATGAG

Sirt2_∆N2_R

5’- CTGAAAATACAGGTTTTCGG

3. QC_Sirt2_∆C_F

5’- CGATTGATGCCCAATCCTGAGCCGGCGTGCCTAATCC

QC_Sirt2_∆C_R

5’- GGATTAGGCACGCCGGCTCAGGATTGGGCATCAATCG

4. Sirt2_A31C-389_F

GCAGGTGGGGAGGCG

Sirt2_A31C-389_R

GCACTGAAAATACAGGTTTTCG

5. Sirt2_A31C-356

Used the same QC primer pair from 3.

**Plasmids and constructs**

1. *His6-SUMO-Sirt2-∆CN (aa 56-356)* plasmid was a gift of Hening Lin lab (pET28a, Kanamycin resistant).

His-SUMO-Sirt2-∆CN (aa 56-356) sequence (His tag is in *italics* SUMO sequence underlined)

*CATCATCATCATCATCAC*AGCAGCGGCCTGGTGCCGCGCGGCAGCCATATGGCTAGCATGTCGGACTCAGAAGTCAATCAAGAAGCTAAGCCAGAGGTCAAGCCAGAAGTCAAGCCTGAGACTCACATCAATTTAAAGGTGTCCGATGGATCTTCAGAGATCTTCTTCAAGATCAAAAAGACCACTCCTTTAAGAAGGCTGATGGAAGCGTTCGCTAAAAGACAGGGTAAGGAAATGGACTCCTTAAGATTCTTGTACGACGGTATTAGAATCCAAGCTGATCAGACCCCTGAAGATTTGGACATGGAGGATAACGATATTATTGAGGCTCACAGAGAACAGATTGGTGGATCCGAGCGTCTGCTGGACGAGCTGACCTTGGAAGGGGTGGCCCGGTACATGCAGAGCGAACGCTGTCGCAGAGTCATCTGTTTGGTGGGAGCTGGAATCTCCACATCCGCAGGCATCCCCGACTTTCGCTCTCCATCCACCGGCCTCTATGACAACCTAGAGAAGTACCATCTTCCCTACCCAGAGGCCATCTTTGAGATCAGCTATTTCAAGAAACATCCGGAACCCTTCTTCGCCCTCGCCAAGGAACTCTATCCTGGGCAGTTCAAGCCAACCATCTGTCACTACTTCATGCGCCTGCTGAAGGACAAGGGGCTACTCCTGCGCTGCTACACGCAGAACATAGATACCCTGGAGCGAATAGCCGGGCTGGAACAGGAGGACTTGGTGGAGGCGCACGGCACCTTCTACACATCACACTGCGTCAGCGCCAGCTGCCGGCACGAATACCCGCTAAGCTGGATGAAAGAGAAGATCTTCTCTGAGGTGACGCCCAAGTGTGAAGACTGTCAGAGCCTGGTGAAGCCTGATATCGTCTTTTTTGGTGAGAGCCTCCCAGCGCGTTTCTTCTCCTGTATGCAGTCAGACTTCCTGAAGGTGGACCTCCTCCTGGTCATGGGTACCTCCTTGCAGGTGCAGCCCTTTGCCTCCCTCATCAGCAAGGCACCCCTCTCCACCCCTCGCCTGCTCATCAACAAGGAGAAAGCTGGCCAGTCGGACCCTTTCCTGGGGATGATTATGGGCCTCGGAGGAGGCATGGACTTTGACTCCAAGAAGGCCTACAGGGACGTGGCCTGGCTGGGTGAATGCGACCAGGGCTGCCTGGCCCTTGCTGAGCTCCTTGGATGGAAGAAGGAGCTGGAGGACCTTGTCCGGAGGGAGCACGCCAGCATAGATGCCCAGTCGTAA

His-SUMO-Sirt2-∆CN (aa 56-356) amino acid sequence (His tag is in *italics* SUMO sequence underlined)

*HHHHHH*SSGLVPRGSHMASMSDSEVNQEAKPEVKPEVKPETHINLKVSDGSSEIFFKIKKTTPLRRLMEAFAKRQGKEMDSLRFLYDGIRIQADQTPEDLDMEDNDIIEAHREQIGGSERLLDELTLEGVARYMQSERCRRVICLVGAGISTSAGIPDFRSPSTGLYDNLEKYHLPYPEAIFEISYFKKHPEPFFALAKELYPGQFKPTICHYFMRLLKDKGLLLRCYTQNIDTLERIAGLEQEDLVEAHGTFYTSHCVSASCRHEYPLSWMKEKIFSEVTPKCEDCQSLVKPDIVFFGESLPARFFSCMQSDFLKVDLLLVMGTSLQVQPFASLISKAPLSTPRLLINKEKAGQSDPFLGMIMGLGGGMDFDSKKAYRDVAWLGECDQGCLALAELLGWKKELEDLVRREHASIDAQS

2. The full-length Sirt2 (aa2-389) was obtained from IDT in the pUCIDT-AMP GoldenGate vector and cloned into a pET28a plasmid (kanamycin resistant). A SUMO sequence was inserted to enhance solubility, and a TEV site was introduced between the SUMO sequence and the Sirt2 sequence. An N-terminal Cys residue was retained for use in potential future chemical modifications.

*His-SUMO-TEV-Sirt2-FL (aa 2-389)* sequence (His tag is in *italics* SUMO sequence underlined and TEV sequence in **bold**)

*CATCATCATCATCATCAC*AGCAGCGGCCTGGTGCCGCGCGGCAGCCATATGGCTAGCATGTCGGACTCAGAAGTCAATCAAGAAGCTAAGCCAGAGGTCAAGCCAGAAGTCAAGCCTGAGACTCACATCAATTTAAAGGTGTCCGATGGATCTTCAGAGATCTTCTTCAAGATCAAAAAGACCACTCCTTTAAGAAGGCTGATGGAAGCGTTCGCTAAAAGACAGGGTAAGGAAATGGACTCCTTAAGATTCTTGTACGACGGTATTAGAATCCAAGCTGATCAGACCCCTGAAGATTTGGACATGGAGGATAACGATATTATTGAGGCTCACAGAGAACAGATTGGTGGATCC**GAAAACCTGTATTTTCAG**TGCGCTGAGCCCGACCCCTCCCATCCTCTTGAGACGCAGGCGGGGAAAGTGCAAGAAGCTCAAGATAGCGATAGCGACAGCGAAGGAGGTGCAGCAGGTGGGGAGGCGGATATGGATTTTCTTCGCAATCTTTTTAGCCAGACTTTAAGTTTGGGTTCTCAGAAAGAGCGTTTGCTTGATGAGTTGACACTGGAAGGTGTAGCACGCTACATGCAAAGTGAACGTTGCCGCCGCGTGATTTGCTTAGTAGGGGCTGGTATCAGCACTAGCGCGGGAATTCCCGATTTTCGTTCCCCATCTACCGGGTTATACGACAATCTTGAAAAATACCACTTGCCATACCCCGAAGCTATCTTTGAGATCTCCTACTTTAAGAAACATCCAGAACCCTTCTTTGCATTAGCGAAAGAACTGTATCCCGGACAATTCAAGCCTACTATCTGTCACTACTTTATGCGTCTTCTGAAAGACAAAGGCTTATTACTGCGCTGTTACACGCAAAACATCGATACGTTGGAACGCATTGCTGGCCTGGAGCAAGAAGACTTGGTGGAAGCTCACGGGACCTTCTACACATCGCACTGTGTCTCAGCGTCCTGTCGTCACGAATATCCACTTTCATGGATGAAAGAAAAGATTTTTTCCGAGGTCACCCCTAAATGTGAAGATTGTCAAAGTTTAGTCAAGCCAGACATCGTGTTCTTTGGGGAAAGCTTGCCTGCACGCTTTTTTAGTTGTATGCAGAGCGACTTCTTAAAGGTAGATTTGTTATTAGTCATGGGAACAAGTCTGCAAGTTCAACCTTTTGCGTCATTAATTTCCAAGGCCCCGCTGTCGACACCGCGCTTACTTATCAACAAGGAAAAGGCCGGTCAGTCAGACCCCTTCCTTGGGATGATTATGGGCCTTGGGGGTGGTATGGATTTTGACAGTAAAAAAGCATACCGCGATGTCGCCTGGTTAGGTGAGTGTGACCAGGGCTGTCTGGCTTTGGCGGAGCTTTTGGGCTGGAAAAAAGAATTGGAGGACCTTGTACGTCGTGAGCACGCTTCGATTGATGCCCAATCCGGTGCCGGCGTGCCTAATCCGAGTACGTCGGCTTCGCCGAAGAAGTCCCCTCCTCCAGCCAAAGACGAGGCTCGCACGACCGAACGTGAGAAGCCTCAGTGA

His-SUMO-TEV-Sirt2-FL (aa 2-389) amino acid sequence (His tag is in *italics* SUMO sequence underlined and TEV sequence in **bold**)

*HHHHHH*SSGLVPRGSHMASMSDSEVNQEAKPEVKPEVKPETHINLKVSDGSSEIFFKIKKTTPLRRLMEAFAKRQGKEMDSLRFLYDGIRIQADQTPEDLDMEDNDIIEAHREQIGGS**ENLYFQ**CAEPDPSHPLETQAGKVQEAQDSDSDSEGGAAGGEADMDFLRNLFSQTLSLGSQKERLLDELTLEGVARYMQSERCRRVICLVGAGISTSAGIPDFRSPSTGLYDNLEKYHLPYPEAIFEISYFKKHPEPFFALAKELYPGQFKPTICHYFMRLLKDKGLLLRCYTQNIDTLERIAGLEQEDLVEAHGTFYTSHCVSASCRHEYPLSWMKEKIFSEVTPKCEDCQSLVKPDIVFFGESLPARFFSCMQSDFLKVDLLLVMGTSLQVQPFASLISKAPLSTPRLLINKEKAGQSDPFLGMIMGLGGGMDFDSKKAYRDVAWLGECDQGCLALAELLGWKKELEDLVRREHASIDAQSGAGVPNPSTSASPKKSPPPAKDEARTTEREKPQ

3. Other mutations were generated by Q5 site-directed mutagenesis. The PCR product was treated with KLD (Kinase-Ligase-Dpn1) enzyme mix and transformed into DH5α E. coli cells. Single colonies were picked and grown overnight in 5 mL Luria Bertani (LB) media supplemented with kanamycin (50 µg/mL), then pelleted by microcentrifugation (4000 rpm, 4°C, 5 minutes). Plasmids were obtained using a mini-prep kit (Plasmid miniprep classic, Zymo) and subjected to Sanger sequencing.

*His-SUMO-TEV-Sirt2-∆C (aa 2-356)* amino acid sequence (His tag is in *italics* SUMO sequence is underlined and TEV sequence in **bold**)

*HHHHHH*SSGLVPRGSHMASMSDSEVNQEAKPEVKPEVKPETHINLKVSDGSSEIFFKIKKTTPLRRLMEAFAKRQGKEMDSLRFLYDGIRIQADQTPEDLDMEDNDIIEAHREQIGGS**ENLYFQ**CAEPDPSHPLETQAGKVQEAQDSDSDSEGGAAGGEADMDFLRNLFSQTLSLGSQKERLLDELTLEGVARYMQSERCRRVICLVGAGISTSAGIPDFRSPSTGLYDNLEKYHLPYPEAIFEISYFKKHPEPFFALAKELYPGQFKPTICHYFMRLLKDKGLLLRCYTQNIDTLERIAGLEQEDLVEAHGTFYTSHCVSASCRHEYPLSWMKEKIFSEVTPKCEDCQSLVKPDIVFFGESLPARFFSCMQSDFLKVDLLLVMGTSLQVQPFASLISKAPLSTPRLLINKEKAGQSDPFLGMIMGLGGGMDFDSKKAYRDVAWLGECDQGCLALAELLGWKKELEDLVRREHASIDAQS

4. *His-SUMO-TEV-Sirt2-∆N1 (aa 38-389)* amino acid sequence (His tag is in *italics* SUMO sequence is underlined and TEV sequence in **bold**)

*HHHHHH*SSGLVPRGSHMASMSDSEVNQEAKPEVKPEVKPETHINLKVSDGSSEIFFKIKKTTPLRRLMEAFAKRQGKEMDSLRFLYDGIRIQADQTPEDLDMEDNDIIEAHREQIGGS**ENLYFQ**MDFLRNLFSQTLSLGSQKERLLDELTLEGVARYMQSERCRRVICLVGAGISTSAGIPDFRSPSTGLYDNLEKYHLPYPEAIFEISYFKKHPEPFFALAKELYPGQFKPTICHYFMRLLKDKGLLLRCYTQNIDTLERIAGLEQEDLVEAHGTFYTSHCVSASCRHEYPLSWMKEKIFSEVTPKCEDCQSLVKPDIVFFGESLPARFFSCMQSDFLKVDLLLVMGTSLQVQPFASLISKAPLSTPRLLINKEKAGQSDPFLGMIMGLGGGMDFDSKKAYRDVAWLGECDQGCLALAELLGWKKELEDLVRREHASIDAQSGAGVPNPSTSASPKKSPPPAKDEARTTEREKPQ

5. *His-SUMO-TEV-Sirt2-∆N2 (aa 56-389)* amino acid sequence (His tag is in *italics* SUMO sequence is underlined and TEV sequence in **bold**)

*HHHHHH*SSGLVPRGSHMASMSDSEVNQEAKPEVKPEVKPETHINLKVSDGSSEIFFKIKKTTPLRRLMEAFAKRQGKEMDSLRFLYDGIRIQADQTPEDLDMEDNDIIEAHREQIGGS**ENLYFQ**ERLLDELTLEGVARYMQSERCRRVICLVGAGISTSAGIPDFRSPSTGLYDNLEKYHLPYPEAIFEISYFKKHPEPFFALAKELYPGQFKPTICHYFMRLLKDKGLLLRCYTQNIDTLERIAGLEQEDLVEAHGTFYTSHCVSASCRHEYPLSWMKEKIFSEVTPKCEDCQSLVKPDIVFFGESLPARFFSCMQSDFLKVDLLLVMGTSLQVQPFASLISKAPLSTPRLLINKEKAGQSDPFLGMIMGLGGGMDFDSKKAYRDVAWLGECDQGCLALAELLGWKKELEDLVRREHASIDAQSGAGVPNPSTSASPKKSPPPAKDEARTTEREKPQ

6. *His-SUMO-TEV-Sirt2 A31C-∆N (aa 31-389)* amino acid sequence (His tag is in *italics* SUMO sequence is underlined and TEV sequence in **bold**), used for protein semisynthesis.

*HHHHHH*SSGLVPRGSHMASMSDSEVNQEAKPEVKPEVKPETHINLKVSDGSSEIFFKIKKTTPLRRLMEAFAKRQGKEMDSLRFLYDGIRIQADQTPEDLDMEDNDIIEAHREQIGGS**ENLYFQ**CAGGEADMDFLRNLFSQTLSLGSQKERLLDELTLEGVARYMQSERCRRVICLVGAGISTSAGIPDFRSPSTGLYDNLEKYHLPYPEAIFEISYFKKHPEPFFALAKELYPGQFKPTICHYFMRLLKDKGLLLRCYTQNIDTLERIAGLEQEDLVEAHGTFYTSHCVSASCRHEYPLSWMKEKIFSEVTPKCEDCQSLVKPDIVFFGESLPARFFSCMQSDFLKVDLLLVMGTSLQVQPFASLISKAPLSTPRLLINKEKAGQSDPFLGMIMGLGGGMDFDSKKAYRDVAWLGECDQGCLALAELLGWKKELEDLVRREHASIDAQSGAGVPNPSTSASPKKSPPPAKDEARTTEREKPQ

7. *His-SUMO-TEV-Sirt2 A31C-∆CN (aa 31-356)* amino acid sequence (His tag is in *italics* SUMO sequence is underlined and TEV sequence in **bold**), used for protein semisynthesis.

*HHHHHH*SSGLVPRGSHMASMSDSEVNQEAKPEVKPEVKPETHINLKVSDGSSEIFFKIKKTTPLRRLMEAFAKRQGKEMDSLRFLYDGIRIQADQTPEDLDMEDNDIIEAHREQIGGS**ENLYFQ**CAGGEADMDFLRNLFSQTLSLGSQKERLLDELTLEGVARYMQSERCRRVICLVGAGISTSAGIPDFRSPSTGLYDNLEKYHLPYPEAIFEISYFKKHPEPFFALAKELYPGQFKPTICHYFMRLLKDKGLLLRCYTQNIDTLERIAGLEQEDLVEAHGTFYTSHCVSASCRHEYPLSWMKEKIFSEVTPKCEDCQSLVKPDIVFFGESLPARFFSCMQSDFLKVDLLLVMGTSLQVQPFASLISKAPLSTPRLLINKEKAGQSDPFLGMIMGLGGGMDFDSKKAYRDVAWLGECDQGCLALAELLGWKKELEDLVRREHASIDAQS

**Figure SI**

**
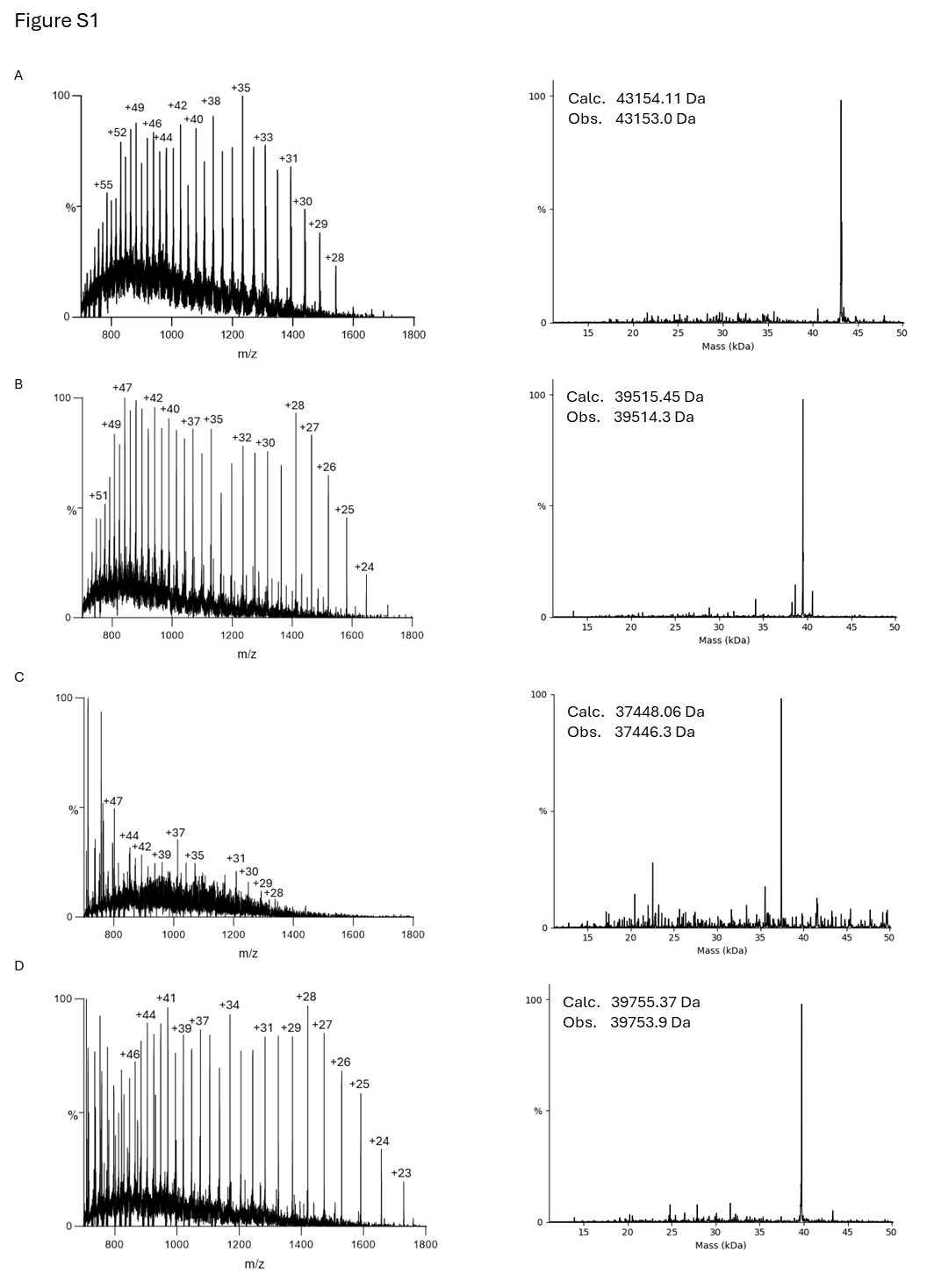
**


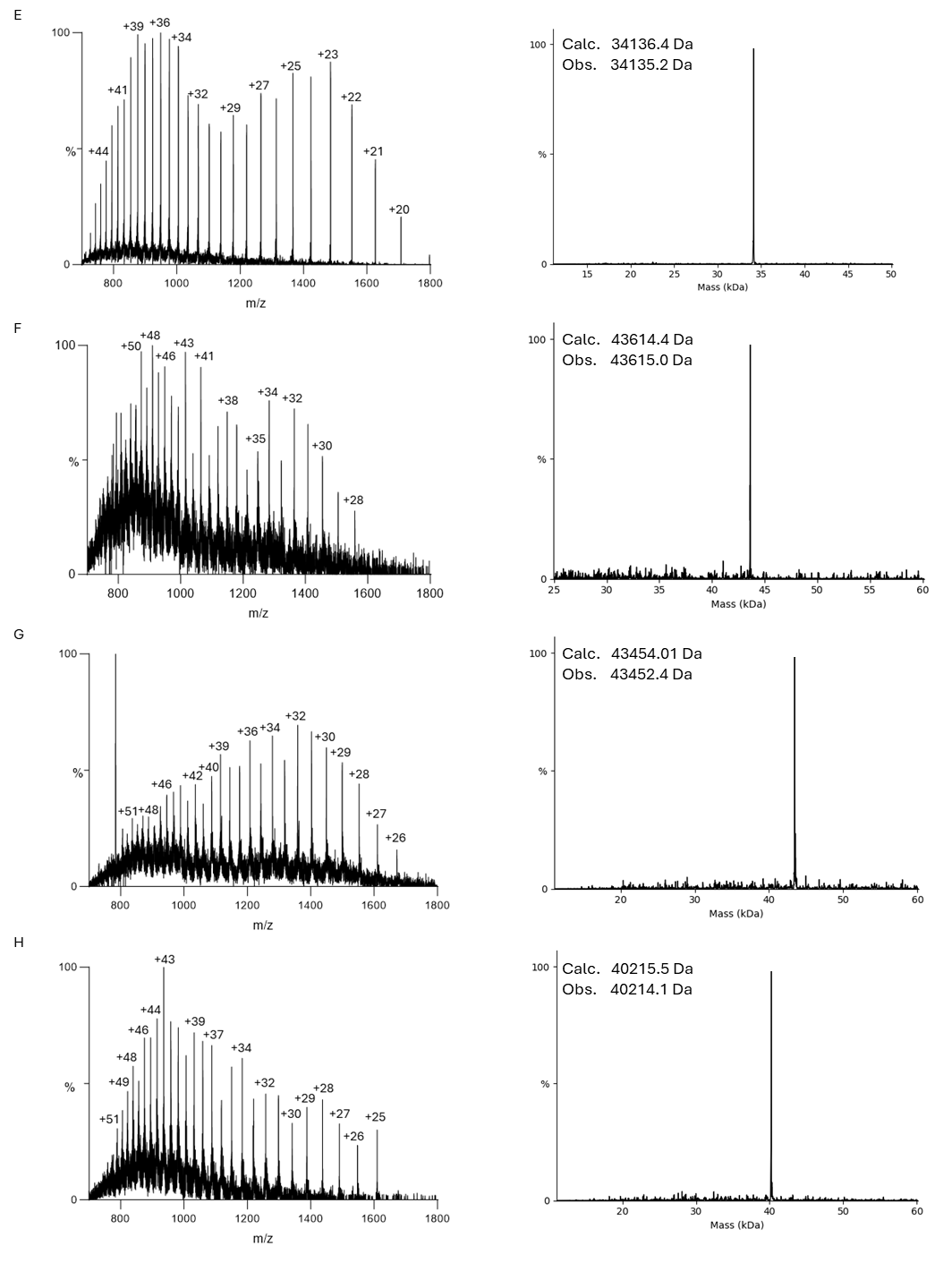


Figure S1. ESI for Sirt2 protein constructs. (A) Intact protein ESI-MS and deconvoluted protein ESI-MS for Sirt2-FL (aa2-389). Calculated average mass for C_1913_H_2991_N_509_O_585_S_21_ [M]^+^: 43154.11 Da; Observed: 43153.0 Da. (B) Intact protein ESI-MS and deconvoluted protein ESI-MS for Sirt2 ∆N1 (aa38-389). Calculated average mass for C_1768_H_2770_N_466_O_520_S_20_ [M]^+^: 39515.45 Da; Observed: 39514.3 Da. (C) Intact protein ESI-MS and deconvoluted protein ESI-MS for Sirt2 ∆N2 (aa56-389). Calculated average mass for C_1676_H_2623_N_441_O_493_S_19_ [M]^+^: 37448.06 Da; Observed 37446.3 Da. (D) Intact protein ESI-MS and deconvoluted protein ESI-MS for Sirt2 ∆C (aa2-356). Calculated average mass for C_1769_H_2754_N_464_O_535_S_21_ [M]^+^: 39755.37 Da; Observed 39753.9 Da. (E) Intact protein ESI-MS and deconvoluted protein ESI-MS for Sirt2 ∆CN (aa56-356). Calculated average mass for C_1535_H_2391_N_397_O_445_S_19_ [M]^+^: 34136.40 Da; Observed 34135.2 Da. (F) Intact protein ESI-MS and deconvoluted protein ESI-MS for diphospho Sirt2-FL. Calculated average mass for C_1926_H_3013_N_511_O_595_P_2_S_22_ [M]^+^: 43614.4 Da; Observed: 43615.0 Da (G) Intact protein ESI-MS and deconvoluted protein ESI-MS for lambda phosphatase treated diphospho Sirt2-FL. Calculated average mass for C_1926_H_3011_N_511_O_589_S_22_ [M]^+^: 43454.01 Da; Observed: 43452.4 Da (H) Intact protein ESI-MS and deconvoluted protein ESI-MS for diphospho Sirt2 ∆C. Calculated average mass for C_1782_H_2776_N_466_O_545_ P_2_S_22_ [M]^+^: 40215.5 Da; Observed: 40214.1 Da.


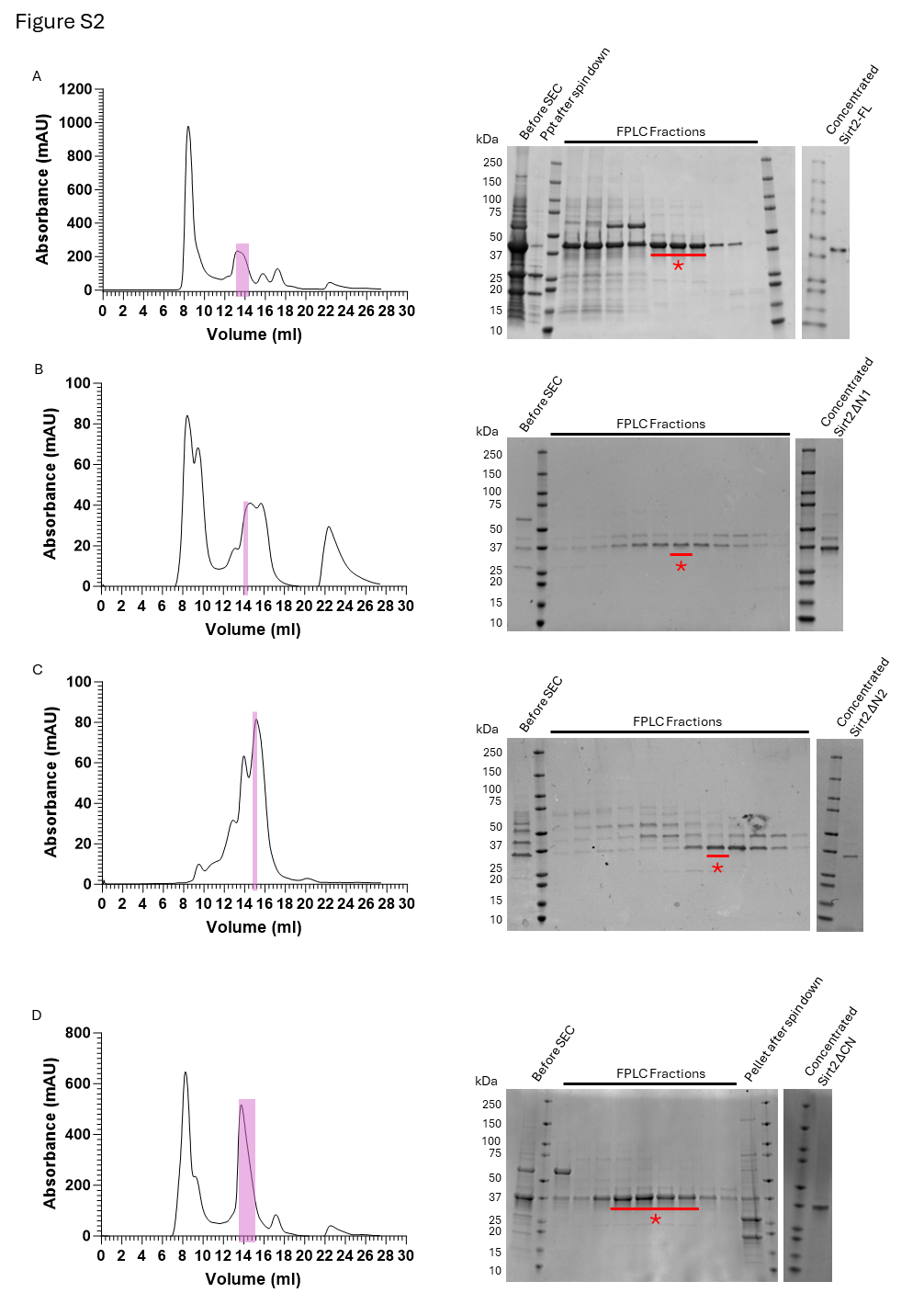


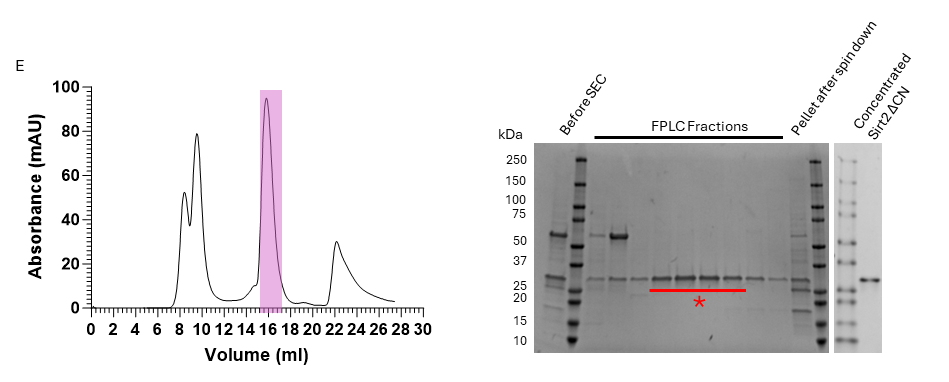


Figure S2. Purification of Sirt2 protein constructs. Superdex200 column FPLC chromatogram and the SDS-PAGE analysis of (A) Sirt2-FL (aa2-389), (B) Sirt2 ∆N1 (aa38-389), (C) Sirt2 ∆N2 (aa56-389), (D) Sirt2 ∆C (aa2-356), (E) Sirt2 ∆CN (aa56-356) purification. Peaks highlighted in pink on the chromatograms/ the fractions marked with an asterisk on the gels, were concentrated and used for the assays.


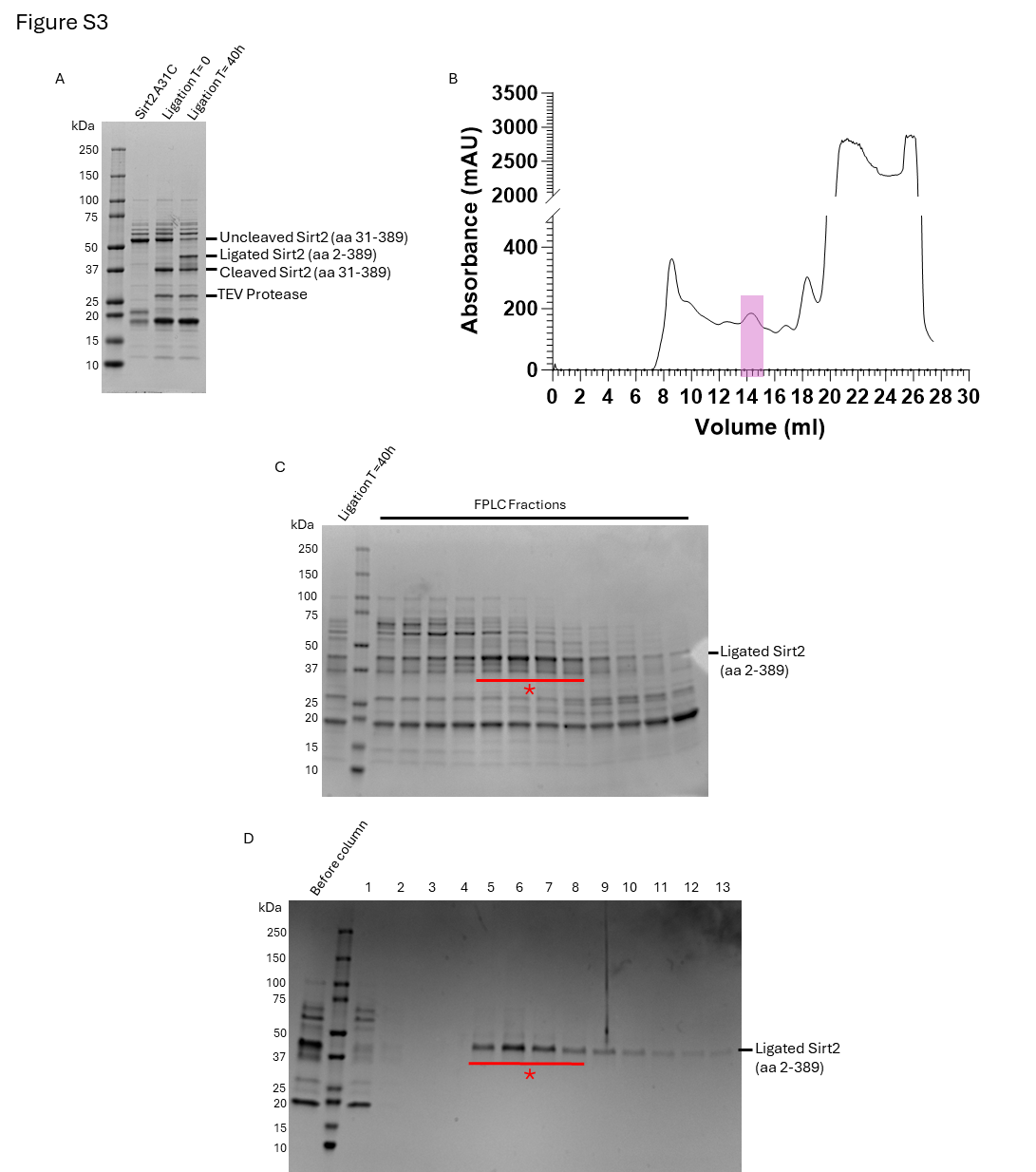


Figure S3. Ligation and purification of diphosphorylated Sirt2-FL. (A) SDS-PAGE analysis of the ligation. (B) Size-exclusion chromatogram of ligated protein. (C) SDS-PAGE analysis of size-exclusion purification of ligated diphosphorylated Sirt2-FL protein. Fractions highlighted with an asterisk were combined and loaded onto the mono-avidin column (D) SDS-PAGE analysis of the mono-avidin purification of diphosphorylated Sirt2-FL. Lane 1: flow-through; Lane2: 500mM NaCl wash; Lane 3: 1M NaCl wash; Lane 4: 150 mM NaCl wash; Lane 5-13: 10 mM biotin elution. Fractions marked with an asterisk were combined and concentrated.


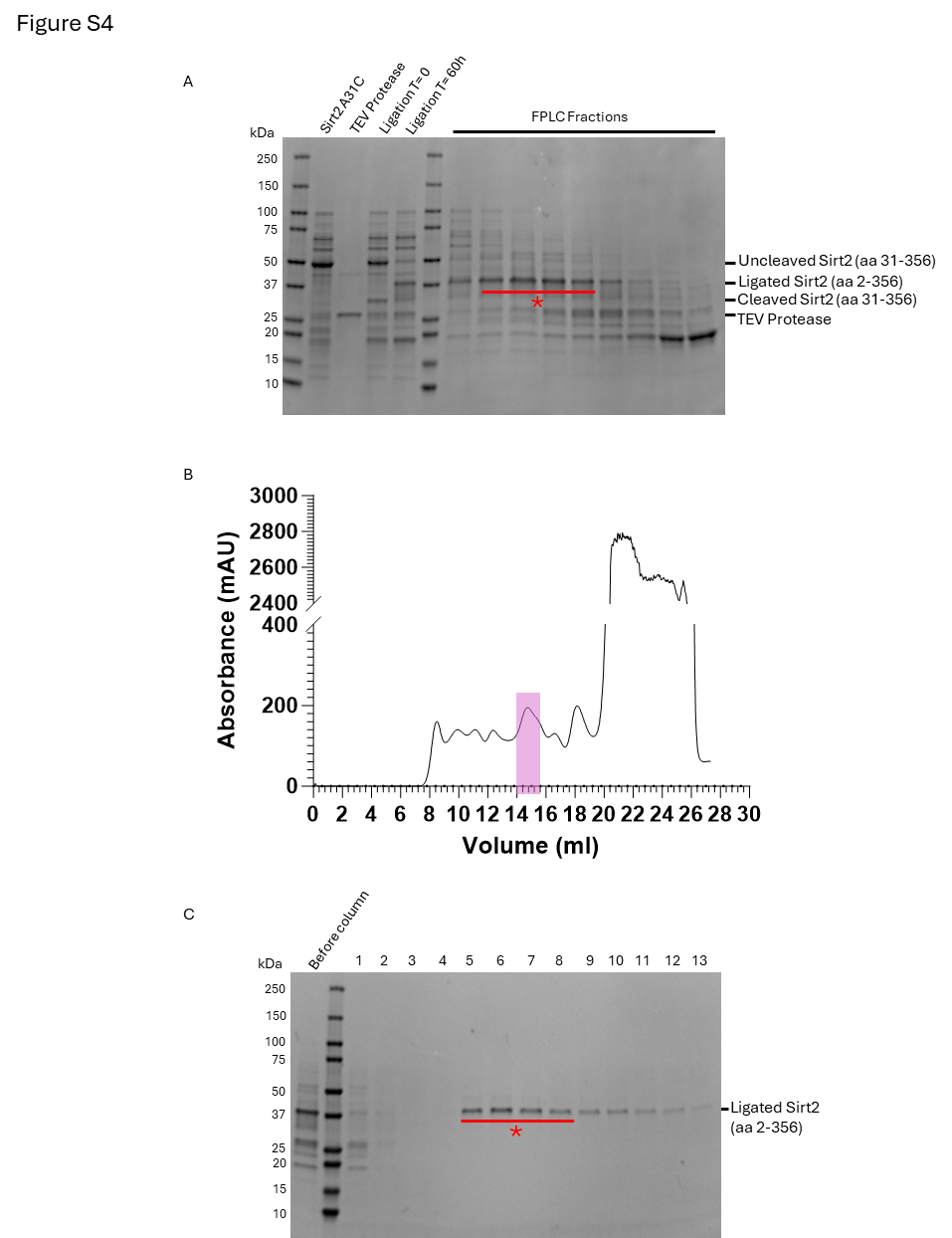


Figure S4. Ligation and purification of diphosphorylated Sirt2 ∆C. (A) SDS-PAGE analysis of ligation and size-exclusion purification of ligated diphosphorylated Sirt2-FL protein. Fractions highlighted with an asterisk were combined and loaded onto the mono-avidin column (B) Size-exclusion chromatogram of ligated protein. (C) SDS-PAGE analysis of the mono-avidin purification of diphosphorylated Sirt2-FL. Lane 1: flow-through; Lane2: 500mM NaCl wash; Lane 3: 1M NaCl wash; Lane 4: 150 mM NaCl wash; Lane 5-13: 10 mM biotin elution. Fractions marked with an asterisk were combined and concentrated.


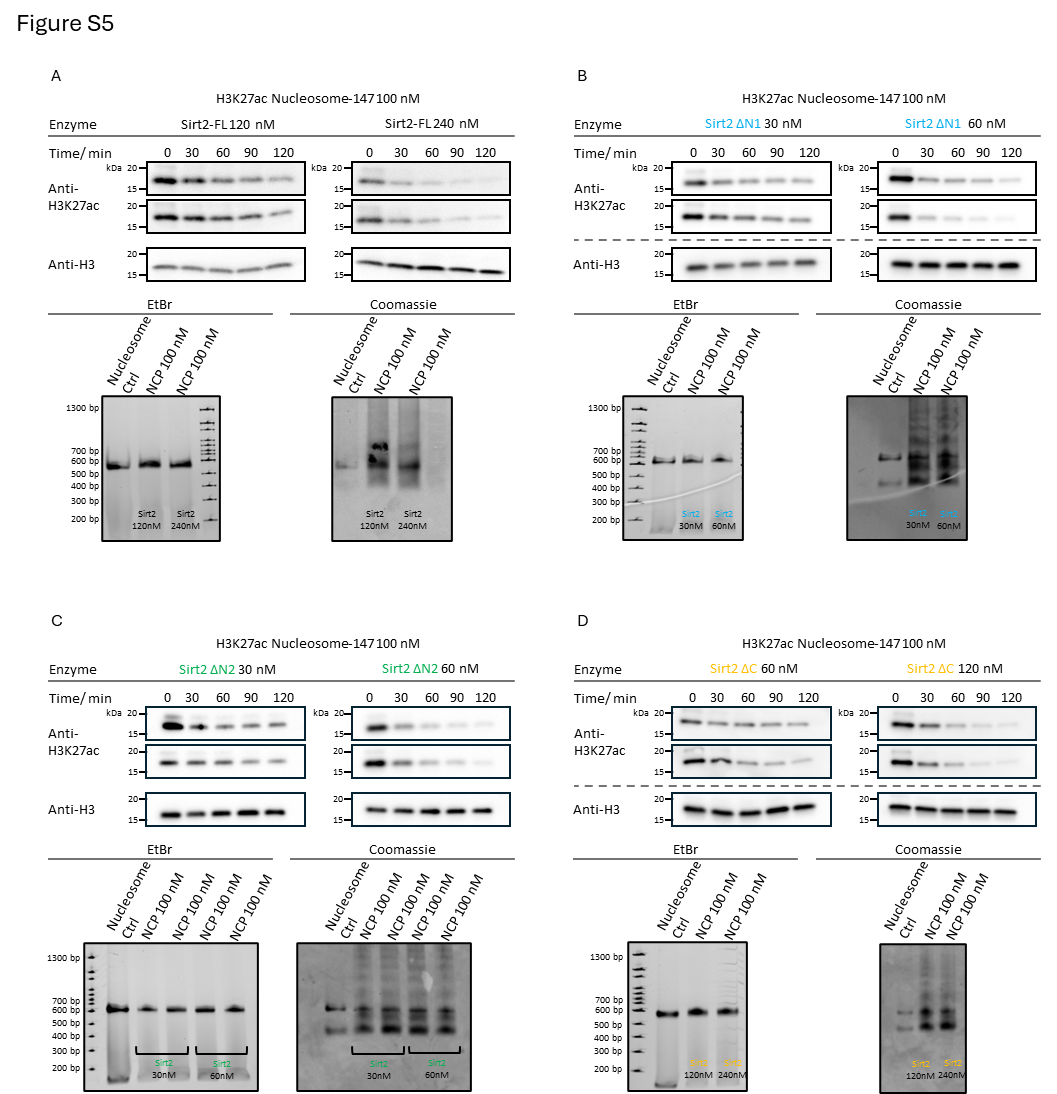


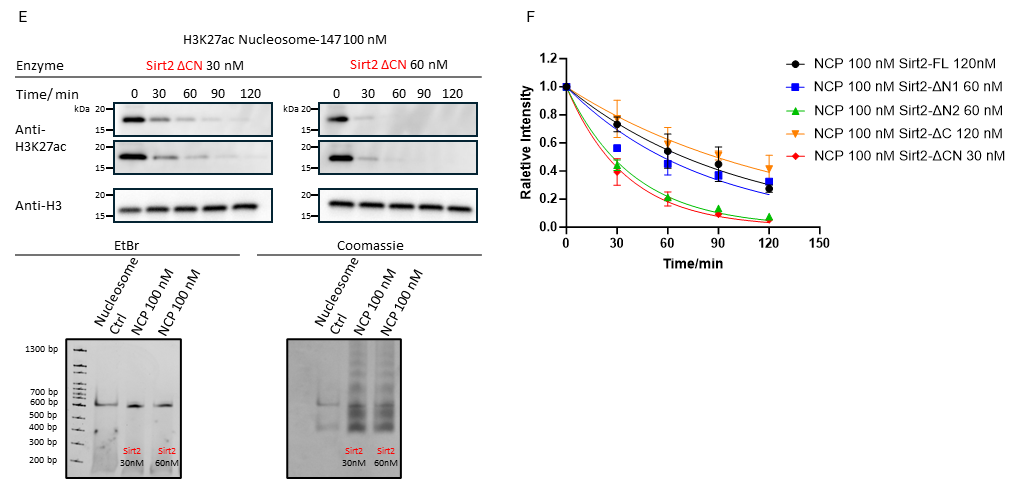


Figure S5. H3K27ac nucleosome deacetylation by different Sirt2 protein constructs. Western blot analysis of H3K27ac nucleosome deacetylation assay by (A) Sirt2-FL (aa2-389), (B) Sirt2 ∆N1 (aa38-389), (C) Sirt2 ∆N2 (aa56-389), (D) Sirt2 ∆C (aa2-356), and (E) Sirt2 ∆CN (aa56-356). (F) Curve fitting for H3K27ac nucleosome kinetics with different Sirt2 protein constructs (black, Sirt2-FL (n=2); blue, Sirt2 ∆N1 (n=2); green, Sirt2 ∆N2 (n=2); orange, Sirt2 ∆C (n=2); red, Sirt2 ∆CN (n=2)). Some figures are reused from Figure 1D.


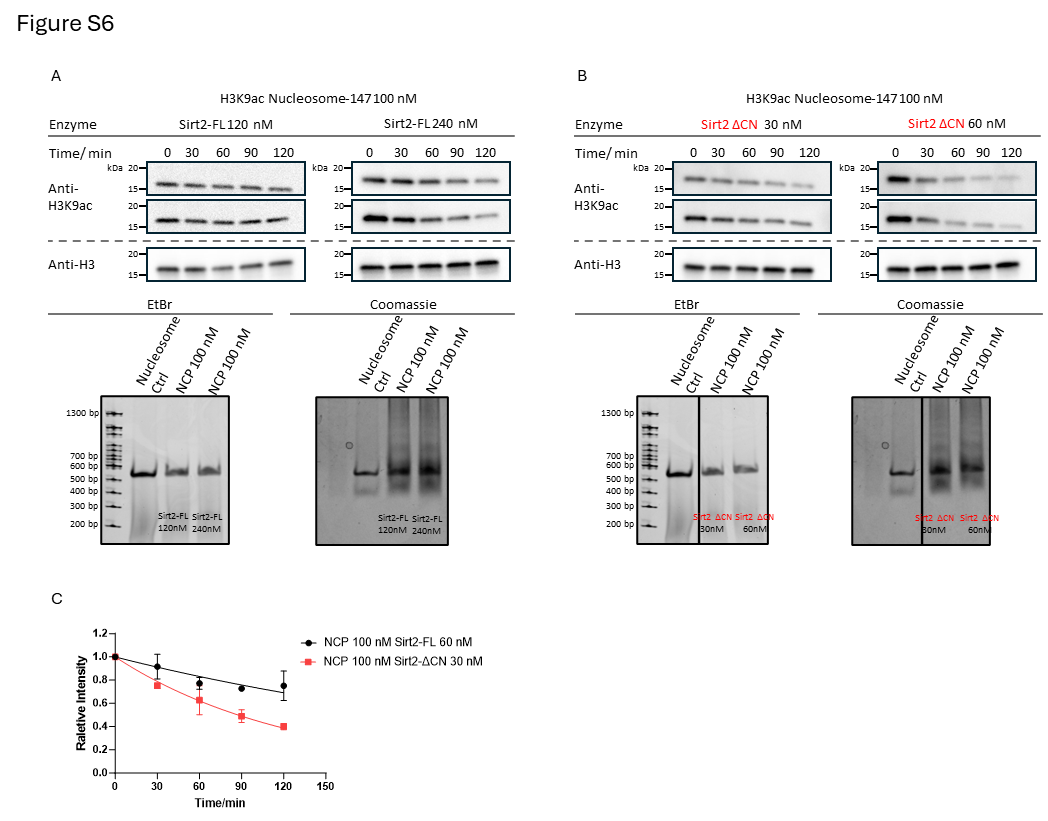


Figure S6. H3K9ac nucleosome deacetylation by different Sirt2 protein constructs. Western blot analysis of H3K9ac nucleosome deacetylation assay by (A) Sirt2-FL (aa2-389) and (B) Sirt2 ∆CN (aa56-356). (C) Curve fitting for H3K9ac nucleosome kinetics with Sirt2-FL and Sirt2 ∆CN (black, Sirt2-FL (n=2); red, Sirt2 ∆CN (n=2)). Note that the marker and nucleosome ctrl lanes in both EtBr and Commassie staining gels are shared by Figure S6A and S6B. Some figures are reused from Figure 1C.


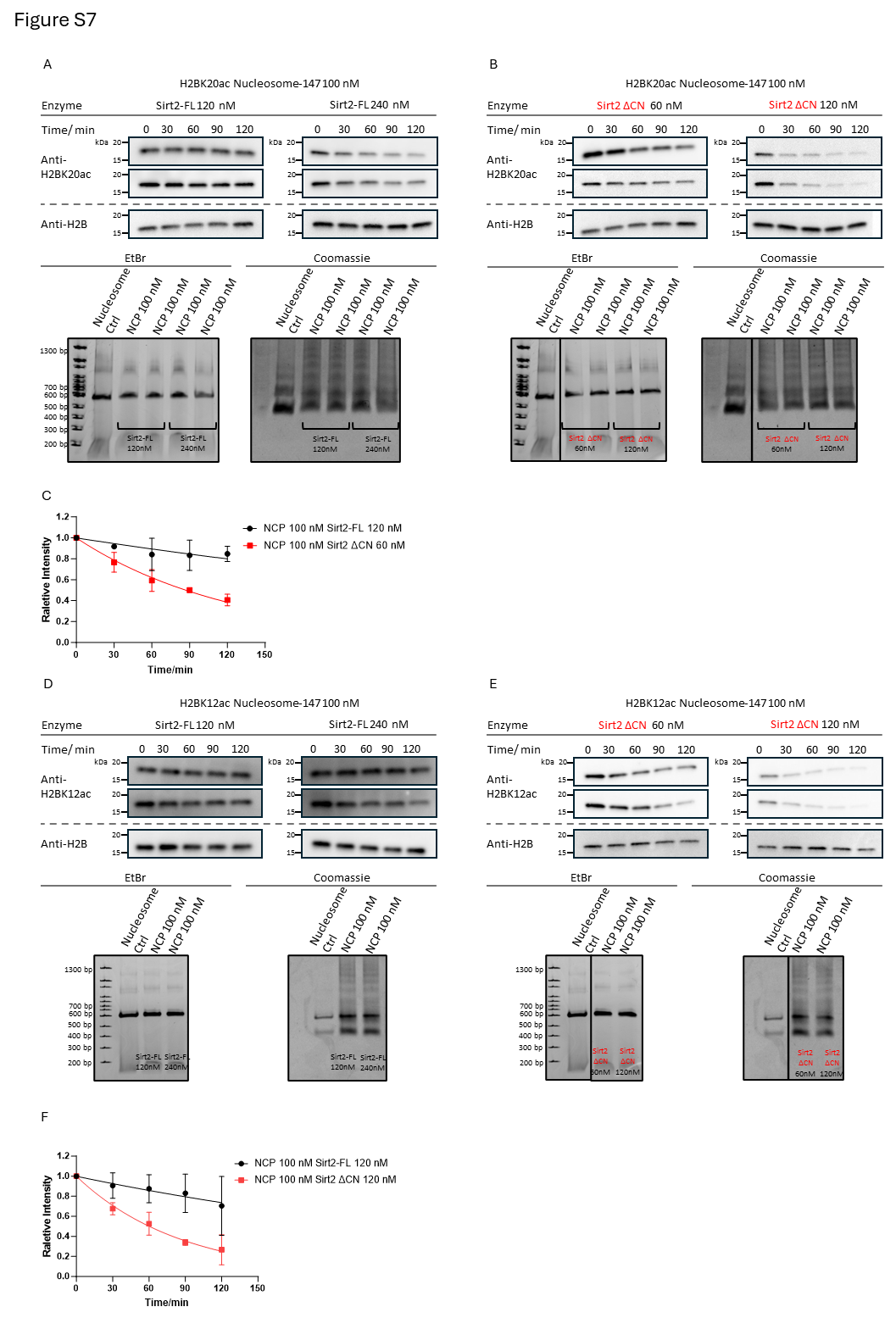


Figure S7. Sirt2 deacetylation on different H2B nucleosome sites. Western blot analysis of H2BK20ac nucleosome deacetylation assay by (A) Sirt2-FL (aa2-389) (B) Sirt2 ∆CN (aa56-356). (C) curve fitting for H2BK20ac nucleosome kinetics with Sirt2-FL and Sirt2 ∆CN (black, Sirt2-FL (n=2); red, Sirt2 ∆CN (n=2)). Western blot analysis of H2BK12ac nucleosome deacetylation assay by (D) Sirt2-FL and (E) Sirt2 ∆CN. (F) Curve fitting for H2BK12ac nucleosome kinetics with Sirt2-FL and Sirt2 ∆CN (black, Sirt2-FL (n=2); red, Sirt2 ∆CN (n=2)). Note that the marker and nucleosome ctrl lanes in both EtBr and Commassie staining gels are shared by Figure S7A and S7B; Figure S7C and S7D. Some figures are reused from Figure 1E,F.


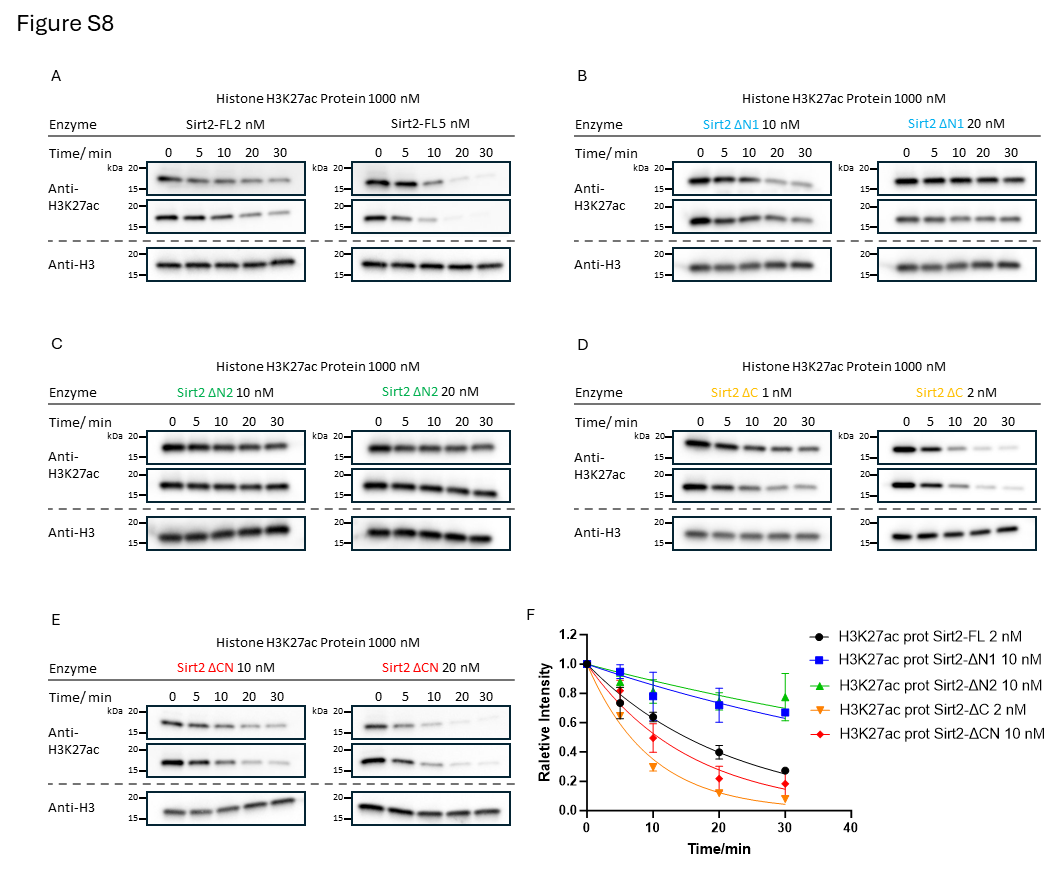


Figure S8. Histone H3K27ac protein deacetylation by different Sirt2 protein constructs. Western blot analysis of histone H3K27ac protein deacetylation assay by (A) Sirt2-FL (aa2-389), (B) Sirt2 ∆N1 (aa38-389), (C) Sirt2 ∆N2 (aa56-389), (D) Sirt2 ∆C (aa2-356), and (E) Sirt2 ∆CN (aa56-356). (F) Curve fitting for H3K27ac nucleosome kinetics with different Sirt2 protein constructs (black, Sirt2-FL (n=2); blue, Sirt2 ∆N1 (n=2); green, Sirt2 ∆N2 (n=2); orange, Sirt2 ∆C (n=2); red, Sirt2 ∆CN (n=2)). Some figures are reused from Figure 2A.


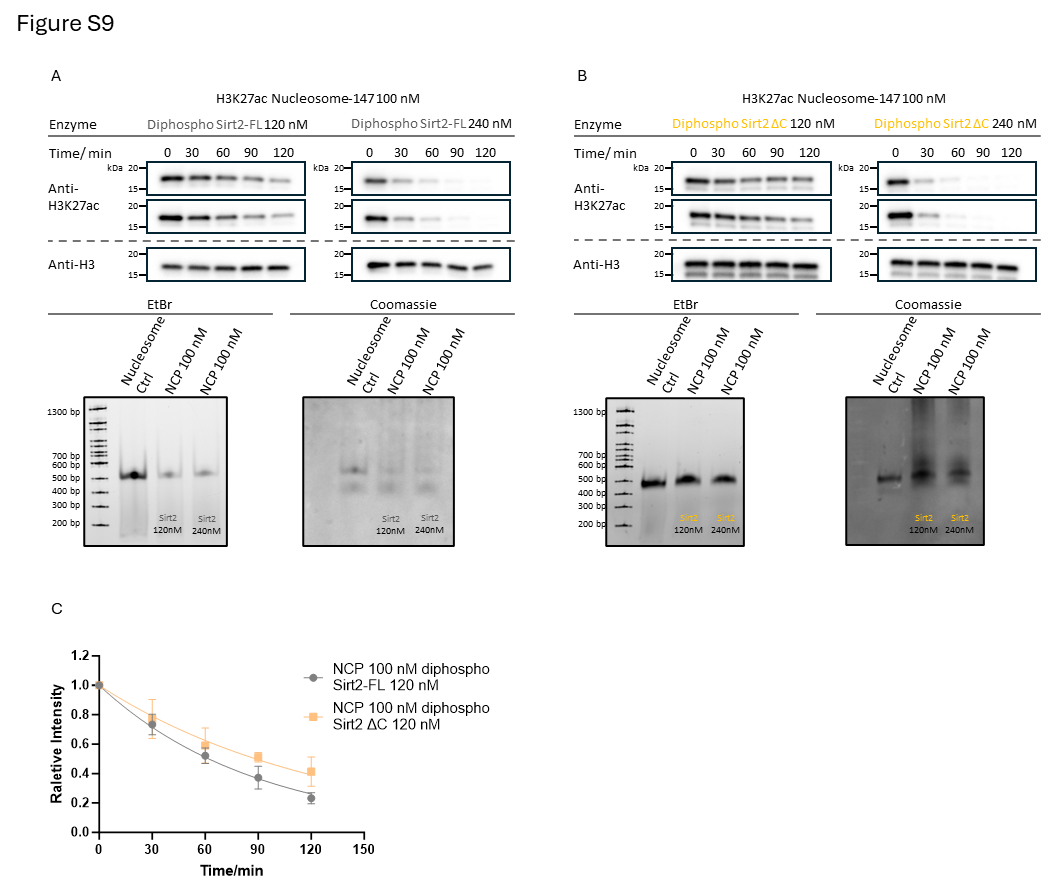


Figure S9. H3K27ac nucleosome deacetylation by diphosphorylated Sirt2 protein constructs. Western blot analysis of H3K27ac nucleosome deacetylation assay by (A) diphospho Sirt2-FL (aa2-389) and (B) diphospho Sirt2 ∆C (aa2-356). (C) Curve fitting for H3K27ac nucleosome kinetics with diphopho Sirt2-FL and diphospho Sirt2 ∆C (grey, diphospho Sirt2-FL (n=2); orange, diphospho Sirt2 ∆C (n=2)). Some figures are reused from Figure 5A.


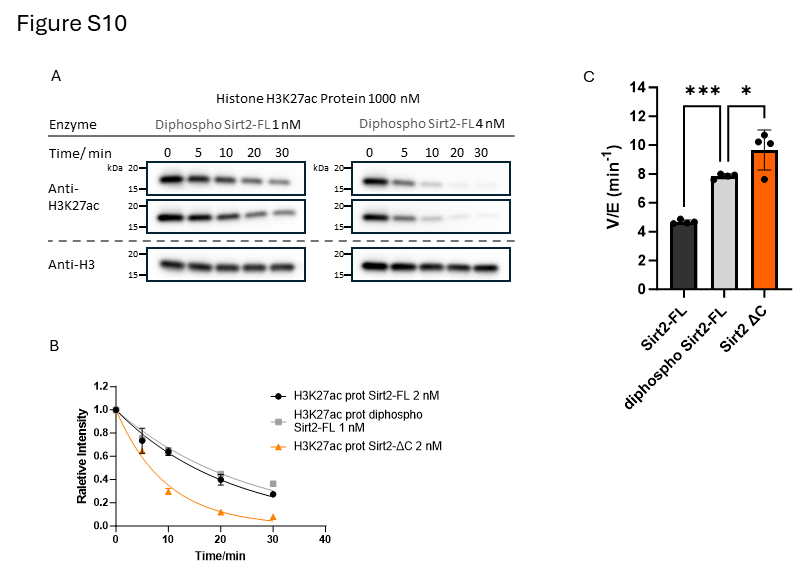


Figure S10. Histone H3K27ac protein deacetylation by diphospho Sirt2-FL protein. (A) Western blot analysis of histone H3K27ac protein deacetylation assay by diphospho Sirt2-FL (B) Curve fitting for H3K27ac nucleosome kinetics with different Sirt2 protein constructs (black, Sirt2-FL; grey, diphospho Sirt2 ∆C (n=2); orange, Sirt2 ∆C (n=2)). (C) Bar graphs representing the comparison of V/[E] for histone H3K27ac protein deacetylation by different Sirt2 protein constructs (dark grey, Sirt2-FL; light grey, diphospho Sirt2 ∆C (n=4); orange, Sirt2 ∆C (n=4)). Statistical analysis was conducted using one way ANOVA followed by Tukey’s post hoc test *P < 0.03, ***P < 0.0002. Some figures are reused from Figure 5B.


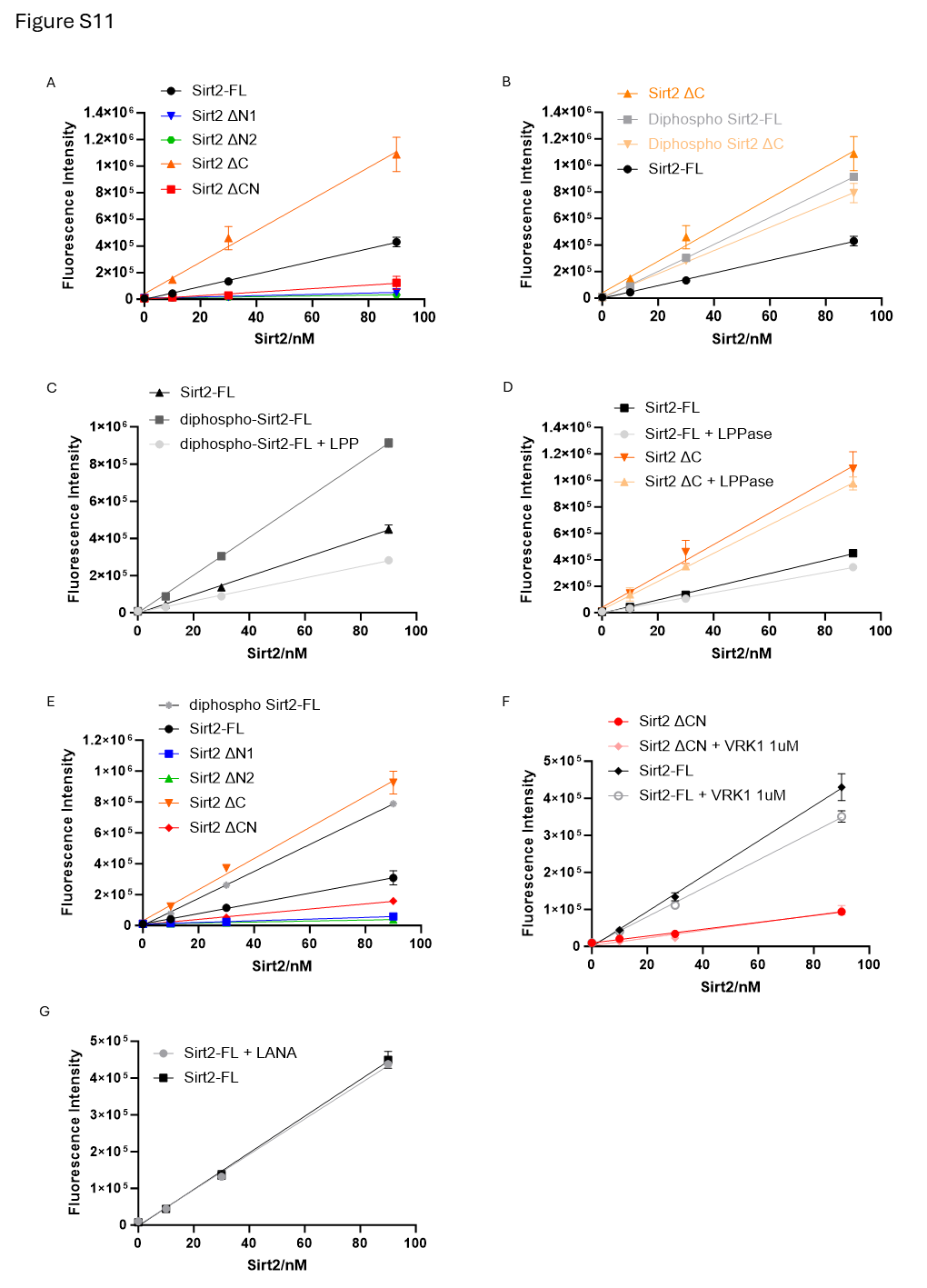


Figure S11. Fluor de Lys assay of acetylated peptide deacetylation by (A) Sirt2 constructs (black: Sirt2-FL (n=6); blue: Sirt2 ∆N1 (n=4); green: Sirt2 ∆N2 (n=2); orange: Sirt2 ∆C (n=4); red: Sirt2 ∆CN (n=4)). (B) unmodified and diphosphorylated Sirt2-FL and Sirt2 ∆C (black: Sirt2-FL (n=6); grey: diphospho Sirt2-FL (n=4); orange: Sirt2 ∆C (n=4); light orange: diphospho Sirt2 ∆C (n=2)). (C) Sirt2-FL (black) (n=6), diphospho Sirt2-FL (dark grey) (n=4) and diphospho Sirt2-FL treated with lambda phosphatase (light grey) (n=2). (D) Sirt2-FL and Sirt2 ∆C with and without lambda phosphatase treatment (black: Sirt2-FL (n=6); grey: Sirt2-FL with lambda phosphatase (n=2); orange: Sirt2 ∆C (n=4); light orange: Sirt2 ∆C with lambda phosphatase (n=2)). (E) Sirt2 constructs at high salt condition (black: Sirt2-FL (n=2); blue: Sirt2 ∆N1 (n=2); green: Sirt2 ∆N2 (n=2); orange: Sirt2 ∆C (n=2); red: Sirt2 ∆CN (n=2); grey: diphospho Sirt2-FL (n=2)). (F) Sirt2-FL with and without VRK1 protein (black: Sirt2-FL (n=6); grey: Sirt2-FL with VRK1, 5mM (n=2); red: Sirt2 ∆CN (n=4); light red: Sirt2 ∆CN with VRK1 (n=2)). (G) Sirt2-FL with and without LANA peptide (black: Sirt2-FL (n=2); grey: Sirt2-FL with LANA peptide, 5mM (n=2)).


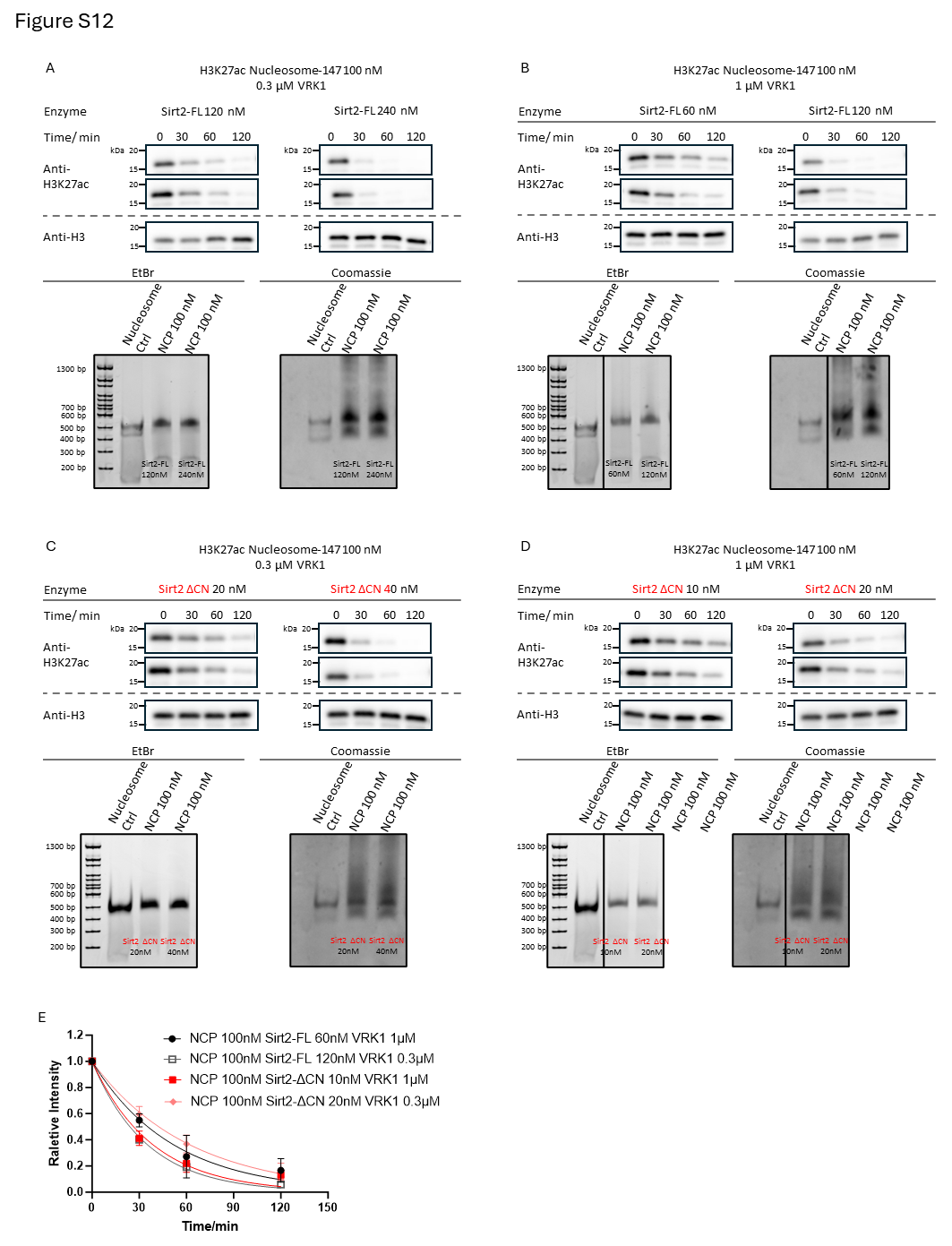
Figure S12. H3K27ac nucleosome deacetylation by different Sirt2 constructs in presence of VRK1 protein. Western blot analysis of deacetylation assay by (A) Sirt2-FL with 0.3 µM VRK1, (B) Sirt2-FL with 1 µM VRK1, (C) Sirt2 ∆CN with 0.3 µM VRK1, (D) Sirt2 ∆CN with 1 µM VRK1. (E) Curve fitting for H3K27ac nucleosome kinetics with Sirt2-FL and Sirt2 ∆CN proteins (black: Sirt2-FL with 1 µM VRK1 (n=2); grey: Sirt2-FL 0.3 µM VRK1 (n=2); red: Sirt2 ∆CN 1 µM VRK1 (n=2); red: Sirt2 ∆CN 1 µM VRK1 (n=2)). Note that the marker and nucleosome ctrl lanes in both EtBr and Commassie staining gels are shared by Figure S12A and S12B, Figure S12C and S12D. Some figures are reused from Figure 3A.


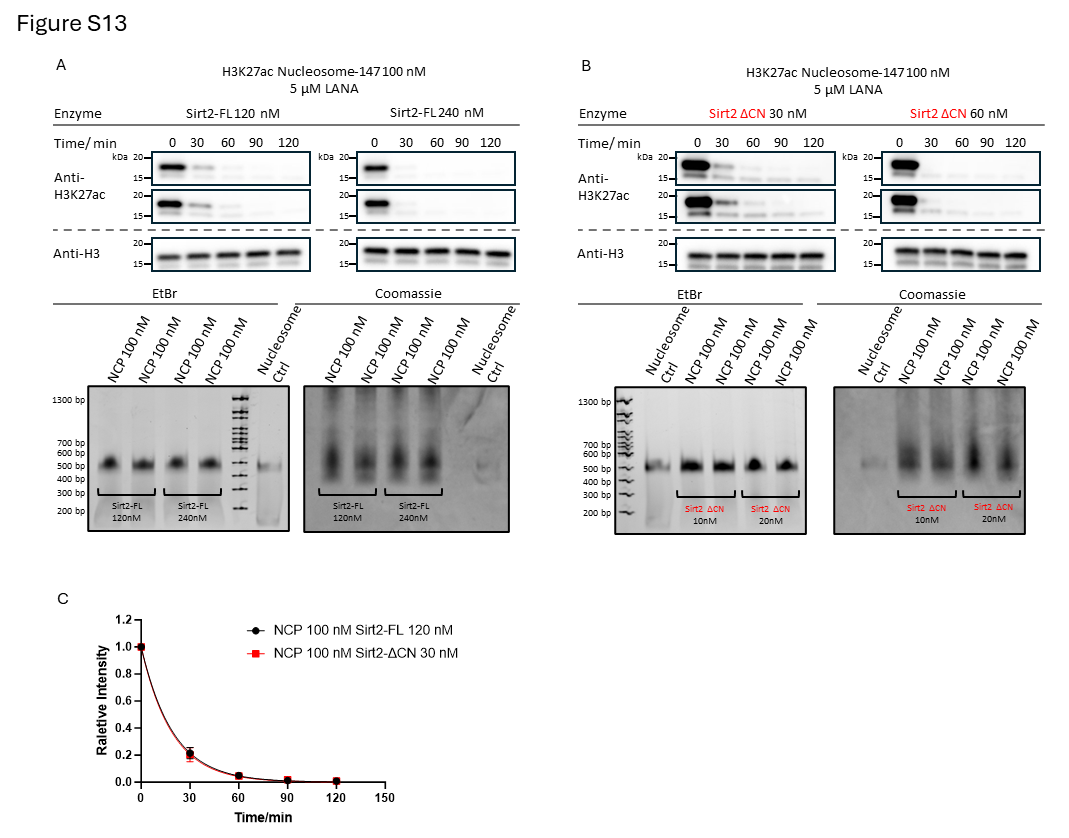


Figure S13. H3K27ac nucleosome deacetylation by different Sirt2 constructs in presence of LANA peptide. Western blot analysis of deacetylation assay in presence of LANA peptide by (D) Sirt2-FL (aa2-389) and (E) Sirt2 ∆CN (aa56-356) proteins. (F) Curve fitting for H3K27ac nucleosome kinetics with Sirt2-FL and Sirt2 ∆CN proteins in presence of LANA peptide (black: Sirt2-FL (n=2); red: Sirt2 ∆CN (n=2)). Some figures are reused from Figure 3B.


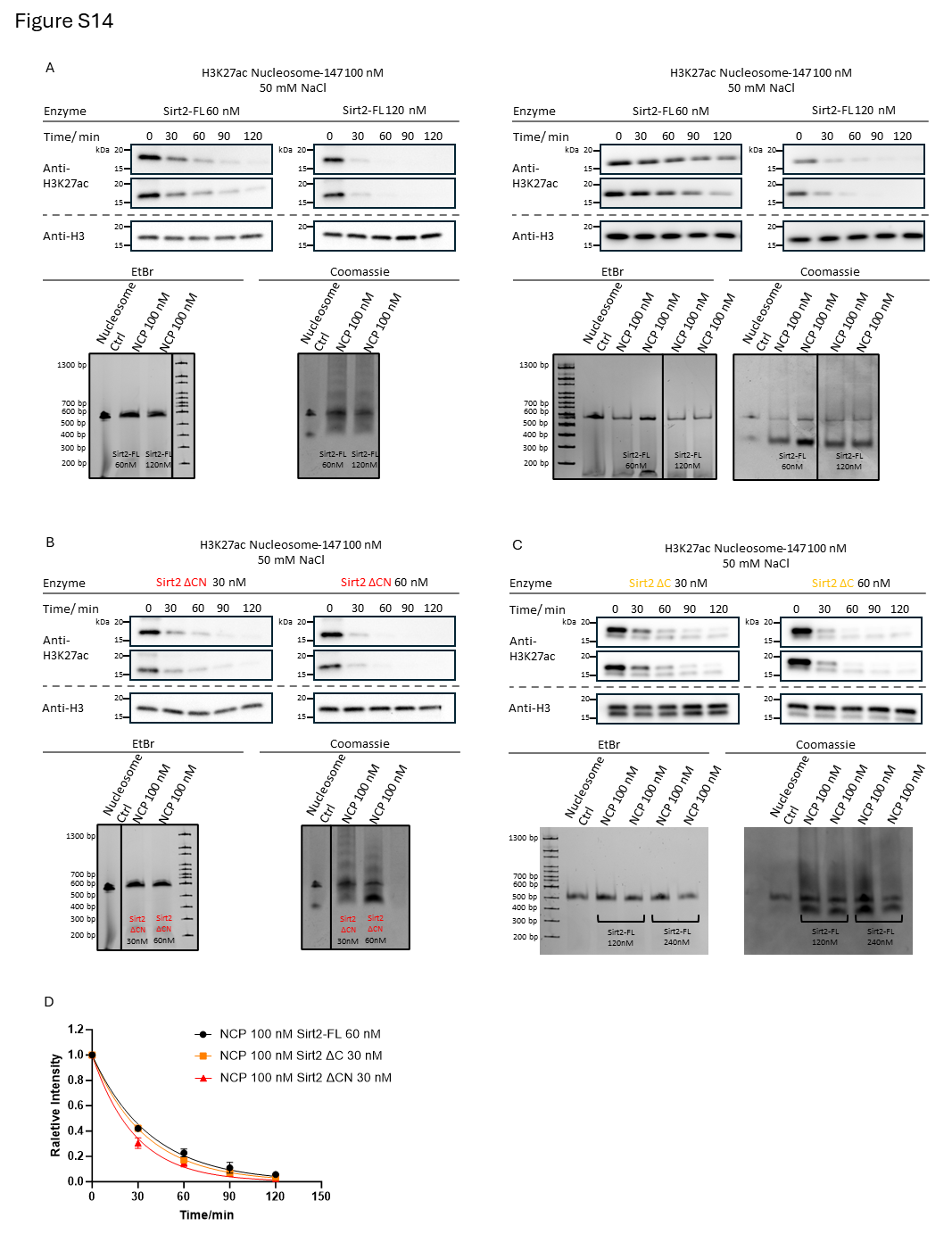


Figure S14. H3K27ac nucleosome deacetylation by different Sirt2 constructs at high salt condition. Western blot analysis of H3K27ac nucleosome deacetylation assay by (A) Sirt2-FL (aa2-389), (B) Sirt2 ∆CN (aa56-356), and (C) Sirt2 ∆C (aa2-356) proteins. (D) Curve fitting for H3K27ac nucleosome kinetics with different Sirt2 protein constructs (black: Sirt2-FL (n=2); orange: Sirt2 ∆C (n=2); and red: Sirt2 ∆CN (n=2)). Note that the marker and nucleosome ctrl lanes in both EtBr and Commassie staining gels are shared by Figure S14A and S14B. Some figures are reused from Figure 7B.


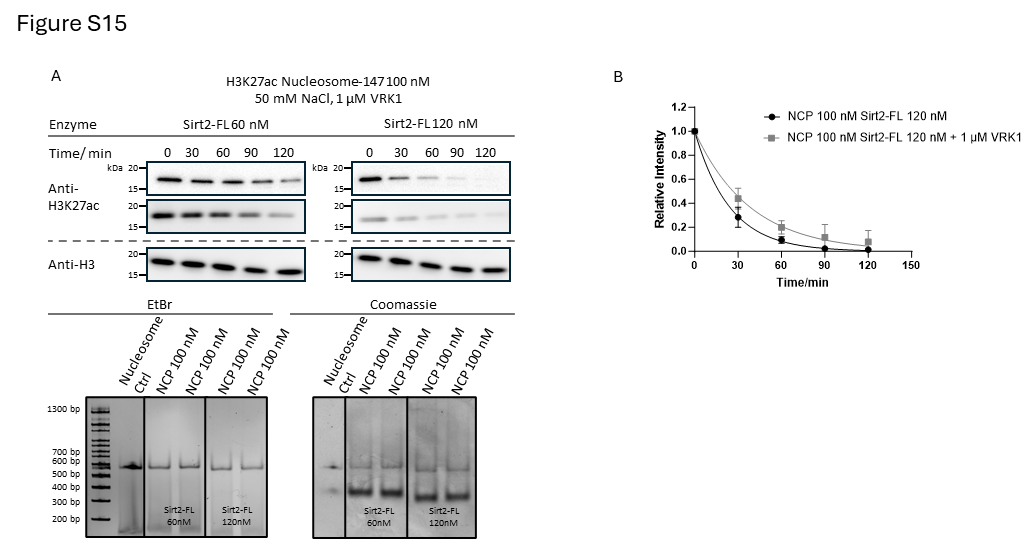


Figure S15. H3K27ac nucleosome deacetylation by Sirt2-FL protein in presence of VRK1 protein at high salt condition. (A) Western blot analysis of H3K27ac nucleosome deacetylation assay by Sirt2-FL protein. (B) Curve fitting for H3K27ac nucleosome kinetics with Sirt2-FL protein with and without VRK1 protein (black: Sirt2-FL (n=2); grey: Sirt2-FL with VRK1 protein (n=2)). Note that the marker and nucleosome ctrl lanes in both EtBr and Commassie staining gels are shared by Figure S14A and S15A. Some figures are reused from Figure 7B.


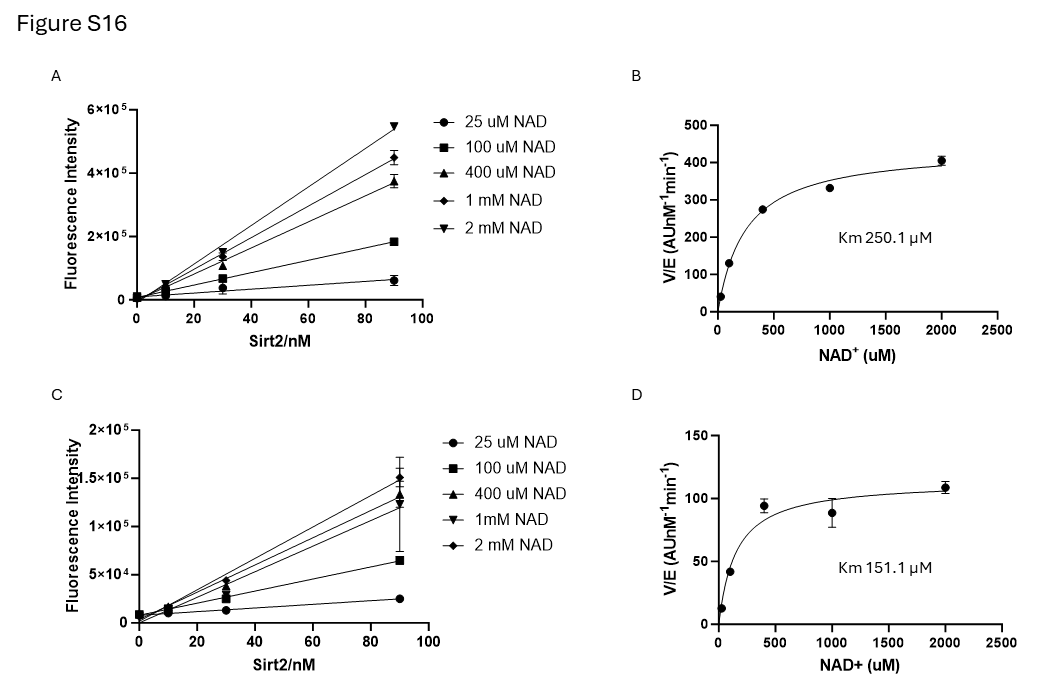


Figure S16. Km of Sirt2-FL and Sirt2 ∆CN for NAD. (A) Fluor de Lys assay of acetylated peptide deacetylation by Sirt2-FL with different NAD concentrations. (B) Michaelis-Menten curve fitting for V/[E] of Sirt2-FL with different concentrations of NAD. (C) Fluor de Lys assay of acetylated peptide deacetylation by Sirt2 ∆CN with different NAD concentrations. (D) Michaelis-Menten curve fitting for V/[E] of Sirt2 ∆CN with different concentrations of NAD. Error bars represent the standard deviation (n=2).


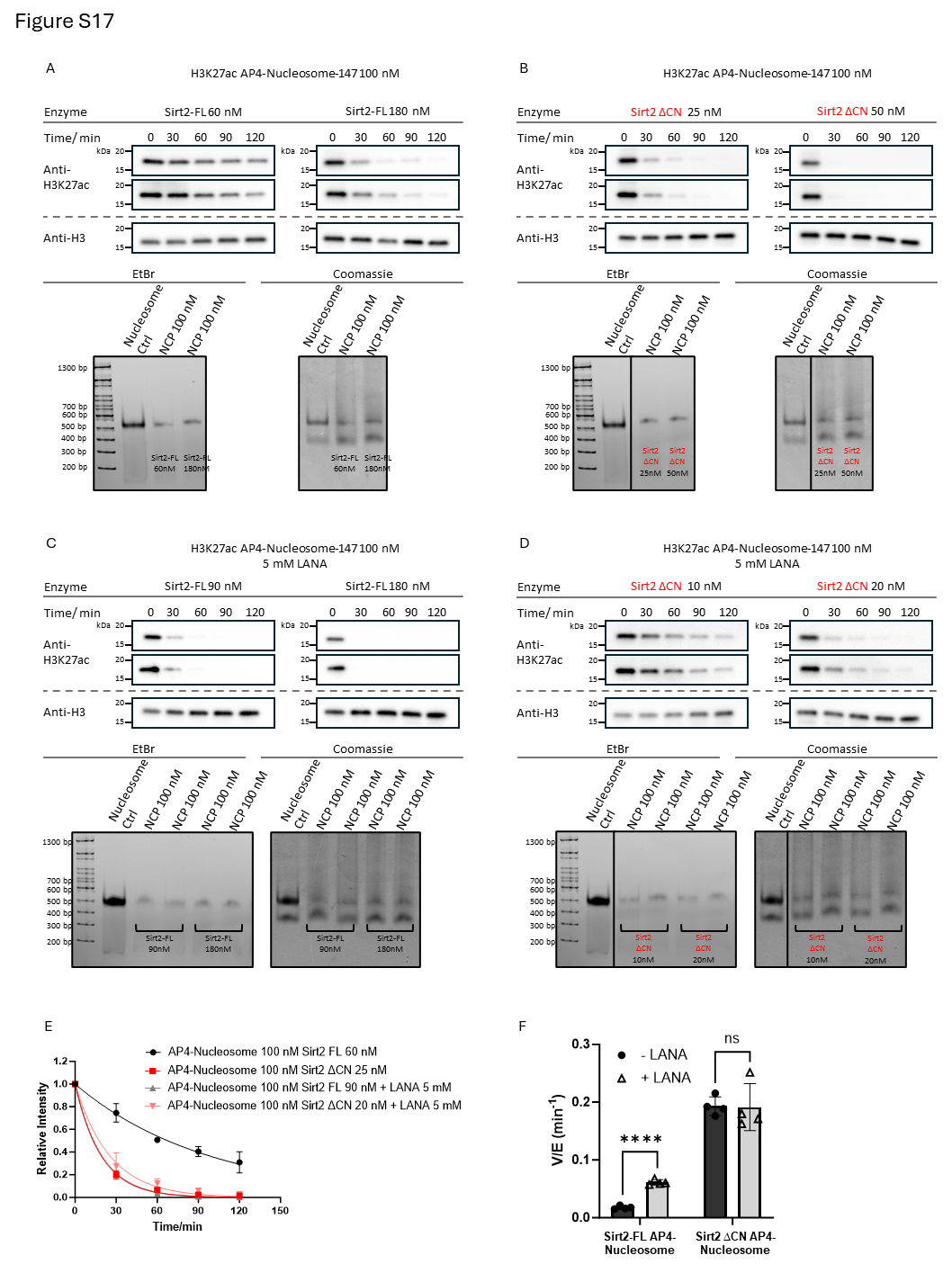


Figure S17. H3K27ac AP4-Nucleosome (H2A E61A/E64A/D90A/E92A) deacetylation by different Sirt2 protein constructs. Western blot analysis of H3K27ac AP4-Nucleosome deacetylation assay by (A) Sirt2-FL (aa2-389) and (B) Sirt2 ∆CN (aa56-356). Western blot analysis of H3K27ac AP4-Nucleosome deacetylation assay in presence of LANA peptide by (C) Sirt2-FL and (D) Sirt2 ∆CN. (E) Curve fitting for H3K27ac AP4-Nucleosome kinetics with different Sirt2 protein constructs (black: Sirt2-FL (n=2); grey: Sirt2 FL + LANA peptide (n=2); red: Sirt2 ∆CN (n=2) and light red: Sirt2 ∆CN + LANA peptide (n=2)). (F) Bar graphs representing the comparison of V/[E] for H3K27ac AP4-Nucleosome deacetylation by different Sirt2 protein constructs. Error bars represent SEM of at least 3 independent experiments. Statistical analysis was conducted using multiple t test, ****P < 0.0001. Note that the marker and nucleosome ctrl lanes in both EtBr and Commassie staining gels are shared by Figure S17A and S17B, Figure S17C and S17D. Some figures are reused from Figure 3C, D.


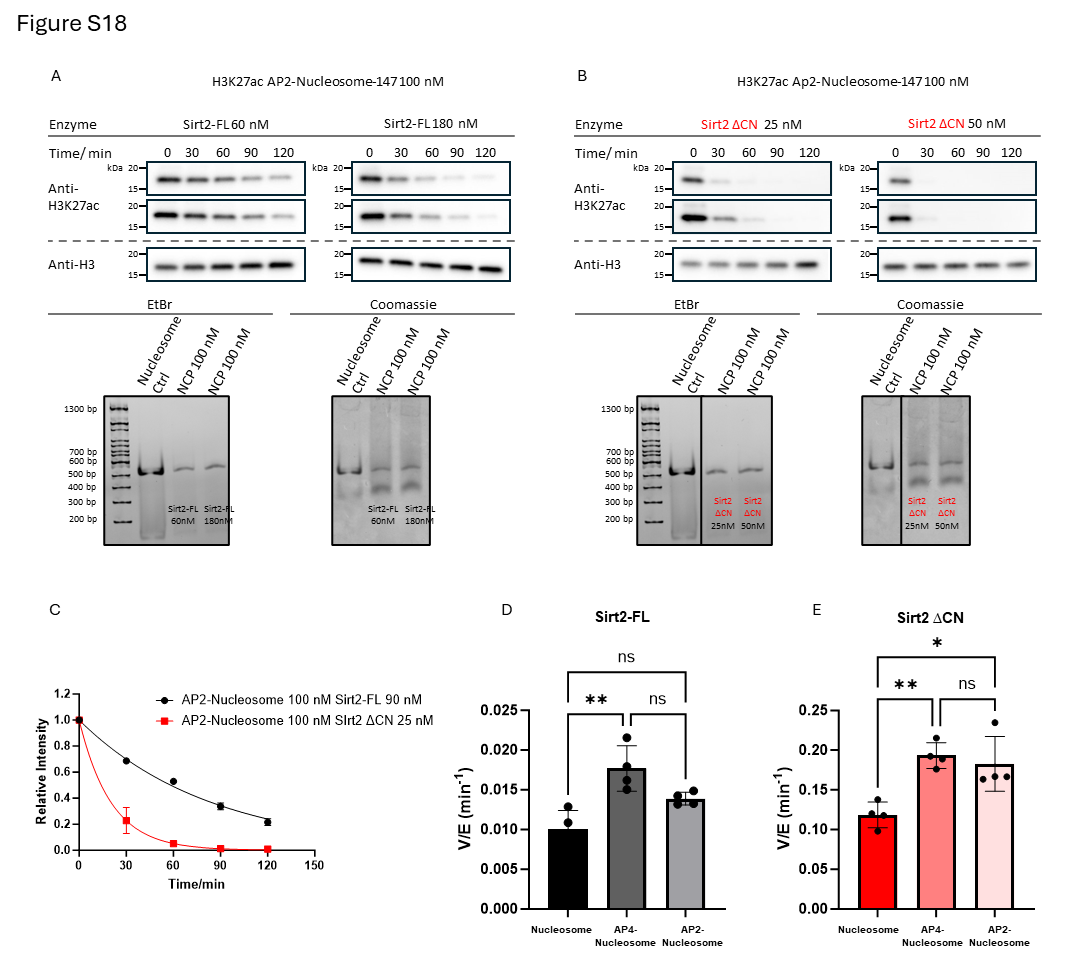


Figure S18. H3K27ac AP2-Nucleosome (H2A D90A/E92A) deacetylation by different Sirt2 protein constructs. Western blot analysis of H3K27ac AP2-Nucleosome deacetylation assay by (A) Sirt2-FL (aa2-389) and (B) Sirt2 ∆CN (aa56-389). (C) Curve fitting for H3K27ac AP2-Nucleosome kinetics with different Sirt2 protein constructs (black: Sirt2-FL (n=2); and red: Sirt2 ∆CN (n=2)). (D) Bar graphs representing the comparison of V/[E] for H3K27ac nucleosome, H3K27ac AP4-Nucleosome and H3K27ac AP2-Nucleosome deacetylation by Sirt2-FL protein. Error bars represent SEM of at least 3 independent experiments. Statistical analysis was conducted using one way ANOVA followed by Tukey’s post hoc test **P < 0.002. (E) Bar graphs representing the comparison of V/[E] for H3K27ac nucleosome, H3K27ac AP4-Nucleosome and H3K27ac AP2-Nucleosome deacetylation by Sirt2 ∆CN protein. Error bars represent SEM of at least 3 independent experiments. Statistical analysis was conducted using one way ANOVA followed by Tukey’s post hoc test *P < 0.03, **P < 0.002. Note that the marker and nucleosome ctrl lanes in both EtBr and Commassie staining gels are shared by Figure S18A and S18B.


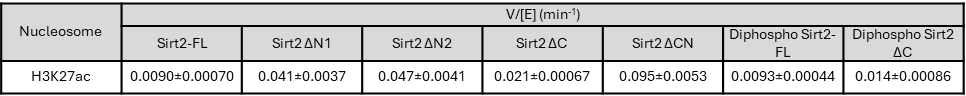


Table S1 Calculated V/[E] for deacetylation of H3K27ac nucleosome by different Sirt2 protein constructs

Table S2 Calculated V/[E] for deacetylation of other nucleosome substrates by Sirt2-FL and Sirt2 ∆CN


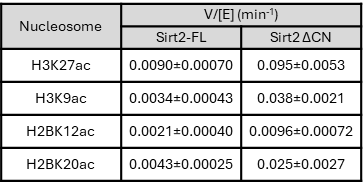


Table S3 Calculated V/[E] for deacetylation of H3K27ac nucleosome by different Sirt2 protein constructs with NaCl, VRK1 and LANA peptide


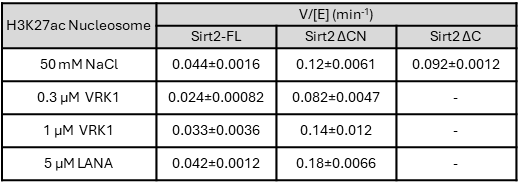


Table S4 Calculated V/[E] for deacetylation of histone H3K27ac protein by different Sirt2 protein constructs


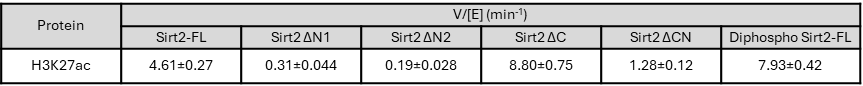


Table S5 Calculated V/[E] for deacetylation of acetylated peptide by different Sirt2 protein constructs


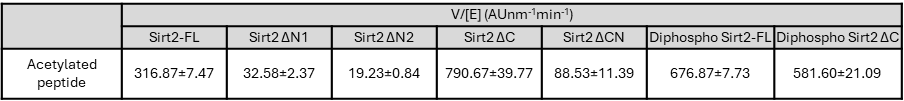


Table S6 Calculated V/[E] for deacetylation of H3K27ac nucleosome, H3K27ac AP4-Nucleosome and H3K27ac AP2-Nucleosome by Sirt2-FL and Sirt2 ∆CN


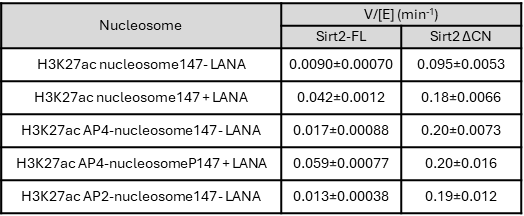

Supplement: Supporting Figures and Tables [file mmc1.docx]
